# VAPPER: High-throughput variant antigen profiling in African trypanosomes of livestock

**DOI:** 10.1093/gigascience/giz091

**Published:** 2019-08-29

**Authors:** Sara Silva Pereira, John Heap, Andrew R Jones, Andrew P Jackson

**Affiliations:** 1 Department of Infection Biology, Institute of Infection and Global Health, University of Liverpool, Liverpool Science Park Ic2, 146 Brownlow Hill, Liverpool L3 5RF, UK; 3 Computational Biology Facility, University of Liverpool, Liverpool L69 7ZB, UK; 4 Institute of Integrative Biology, University of Liverpool, Liverpool L69 7ZB, UK

**Keywords:** VAPPER, variant antigen profiling, African trypanosomes, variant surface glycoproteins

## Abstract

**Background:**

Analysing variant antigen gene families on a population scale is a difficult challenge for conventional methods of read mapping and variant calling due to the great variability in sequence, copy number, and genomic loci. In African trypanosomes, hemoparasites of humans and animals, this is complicated by variant antigen repertoires containing hundreds of genes subject to various degrees of sequence recombination.

**Findings:**

We introduce Variant Antigen Profiler (VAPPER), a tool that allows automated analysis of the variant surface glycoprotein repertoires of the most prevalent livestock African trypanosomes. VAPPER produces variant antigen profiles for any isolate of the veterinary pathogens *Trypanosoma congolense* and *Trypanosoma vivax* from genomic and transcriptomic sequencing data and delivers publication-ready figures that show how the queried isolate compares with a database of existing strains. VAPPER is implemented in Python. It can be installed to a local Galaxy instance from the ToolShed (https://toolshed.g2.bx.psu.edu/) or locally on a Linux platform via the command line (https://github.com/PGB-LIV/VAPPER). The documentation, requirements, examples, and test data are provided in the Github repository.

**Conclusion:**

By establishing two different, yet comparable methodologies, our approach is the first to allow large-scale analysis of African trypanosome variant antigens, large multi-copy gene families that are otherwise refractory to high-throughput analysis.

## Background

Advances in next-generation sequencing have enabled researchers to produce high-throughput genomic data for diverse pathogens. However, analysing multi-copy, contingency gene families remains challenging due to their abundance, high mutation and recombination rates, and unstable gene loci [1]. Yet, these gene families are often involved in many processes of pathogenesis, including antigenic variation, virulence, host use, and immune modulation in a multitude of pathogens [2–4]. A prime example of a crucial gene family lacking the necessary analytic tools for high-throughput analysis is the variant surface glycoprotein (*VSG*) superfamily in African trypanosomes [5].

African trypanosomes are extracellular hemoparasites that cause human sleeping sickness and animal African trypanosomiasis (AAT). Their genomes contain up to 2,500 *VSG* genes [6, 7] dispersed through specialized, hemizygous chromosomal regions called subtelomeres, smaller chromosomes, and less frequently in the core of megabase-sized diploid chromosomes. The *VSG* genes encode variant surface glycoproteins, glycosylphosphatidylinositol-anchored proteins that coat the entire surface of the parasite in the bloodstream of the mammalian host, which function mostly in antigenic variation and immune modulation [8]. Sporadically, specific *VSG* genes have been shown to evolve other functions, not related to antigenic variation, such as conferring human infectivity to *Trypanosoma brucei gambiense* (*TgsGP* gene) [9, 10] and *Trypanosoma brucei rhodesiense* (*SRA* gene) [11, 12], resistance to the drug suramin (*VSG*^sur^ gene) [13], and mediating the transport of transferrin (*TfR* genes) [7, 14].

Because they are key players in host-trypanosome interaction, understanding *VSG* diversity and its impact in pathology, disease phenotype, and virulence is of foremost importance in trypanosome research [4]. However, the *VSG* repertoire cannot be accurately analysed using conventional approaches of read mapping and variant calling. Attempts to bypass this challenge have resulted in alternative approaches using manually-curated *VSG* gene databases for specific *T. brucei* strains [6, 15–17], but to the best of our knowledge there is no automated tool for the systematic analysis of *VSG* from any trypanosome genome. Thus, we have developed Variant Antigen Profiler (VAPPER), a tool that examines *VSG* repertoires in DNA/RNA sequence data of the main livestock trypanosomes, *Trypanosoma congolense* and *Trypanosoma vivax*, and quantifies antigenic diversity. This results in a variant antigen profile (VAP) that can be compared between isolates, locations, and experimental conditions [18].

Studying variant antigen profiles may reveal important aspects of the host-pathogen interaction. For example, we have recently shown that *T. congolense* phylotype 8 transcripts are abundant in metacyclic parasites and that this abundance is attributed to the phylotype in its entirety rather than a specific gene [18]. Similarly, in *Plasmodium falciparum*, Group A *var* genes as a whole, and not individual genes, have been linked to severe disease [19]. Therefore, for some purposes, studying variant antigen profiles can be more informative than individual gene analysis. In this paper, we briefly present how VAPPER can be used to further our knowledge of antigenic diversity and variation.

## Findings

### The service

**Figure 1:**
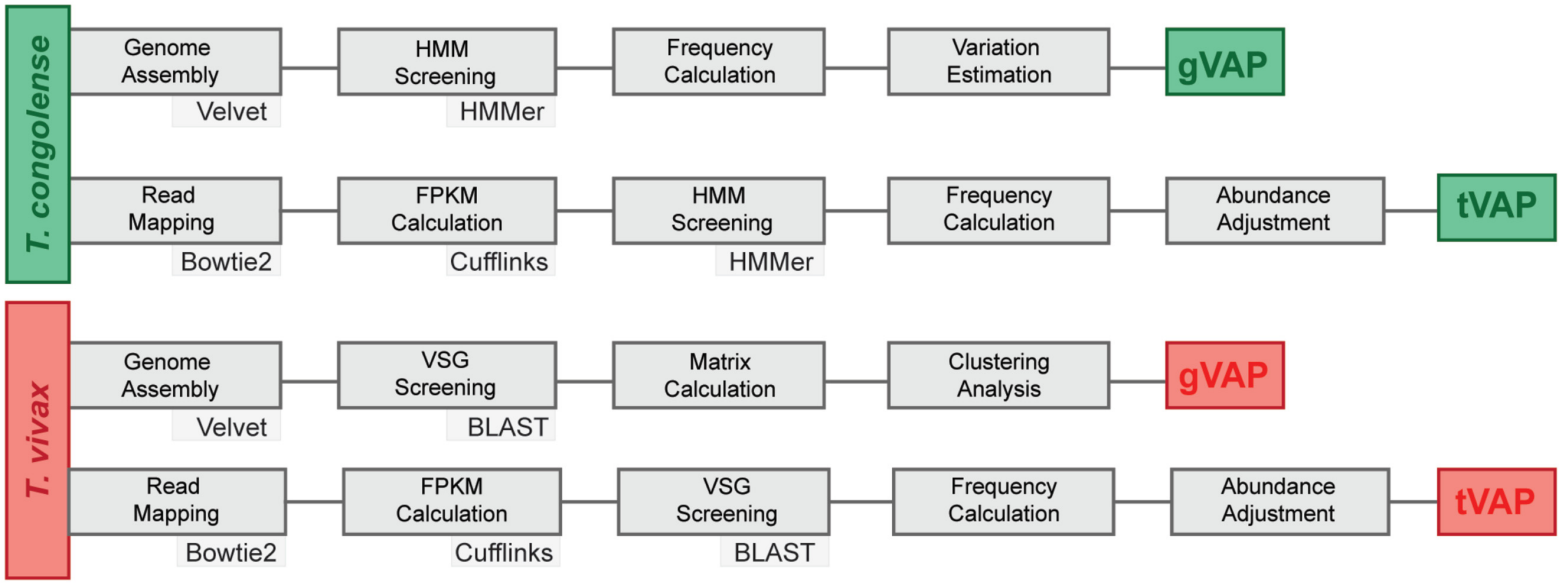
Methodological workflow according to species (*T. congolense* or *T. vivax*) and input data (genomic [gVAP] or transcriptomic [tVAP]).

VAPPER is primarily intended for producing and comparing VAPs of livestock trypanosomes, without the need for complex bioinformatic processes. It is available online through the Galaxy ToolShed [20] for a local Galaxy server [21], and as a Linux package for local installation. The program has 4 pipelines, specific for each organism (*T. congolense* or *T. vivax*) and input data type (genome or transcriptome). VAPPER requires quality-filtered, trimmed, paired sequencing reads in FASTQ format [22] or assembled contigs in FASTA format [23]. Results are presented in tables of frequencies, heat maps, and principal component analysis (PCA) plots, visualized as HTML files or exported to PDF or PNG format. A typical workflow is shown in Fig. 1.

For *T. congolense* genomic VAPs (gVAP), VAPPER starts with genome assembly of raw, short reads using Velvet 1.2.10 [24]. Assembled contigs are screened for predefined protein motifs described by a hidden Markov model (HMM) using HMMER 3.1b2 [25] after 6-frame translation. A detailed description of the universal protein motifs and their biological significance is presented in a recent article [18], but, in summary, each protein motif or motif combination is diagnostic of a specific phylotype [18]; therefore, phylotype frequency can be calculated from the HMMER output. The proportions of each phylotype represent the gVAP and are recorded in a table of frequencies. The gVAP produced is also placed in the context of a *T. congolense* genome database supplied with VAPPER (N = 97 [18, 26]), which is regularly updated. This is achieved through a Euclidean distance-based clustering analysis. Results are presented as 2 heat maps with corresponding dendrograms, one showing phylotype frequency and the other showing frequency deviation from the population mean. They are also shown as a PCA plot and a table of frequencies.

For *T. congolense* transcriptomic analyses (tVAP), VAPPER performs read mapping using Bowtie 2 2.2.6 [27], reference-based transcript assembly and abundance calculation using Cufflinks 2.2.1 [28], and *VSG* transcript screening and phylotype assigning as described for gVAP. The proportions of each phylotype are then adjusted for transcript abundance based on the Cufflinks output. The tVAP is presented as a weighted bar chart and compared to the gVAP of the reference. Ideally, the user would provide their own reference genome for the mapping step. Because that is not always possible, especially for field isolate analysis, we provide 2 reference genomes, the IL3000 Kenyan isolate [7, 29] and the Tc1/148 (MBOI/NG/60/1-148) Nigerian isolate [30, 31]. Choosing the most adequate reference for the sample being analysed may potentially improve VAPPER results by increasing mapping sensitivity. However, we have previously shown that closely related *T. congolense* strains (i.e., with short genetic distances) do not always have equally related *VSG* repertoires [18].

For *T. vivax*, the gVAP is based on the presence or absence of predefined *VSG* genes, rather than the phylotype frequencies described for *T. congolense*. The *T. vivax VSG* repertoire is composed of distantly related lineages with sequence diversity as low as 40% [7]. These lineages are broadly conserved across isolates, which allows us to build a *VSG* database for the entire species. *VSG*-containing contigs are identified using BLAST 2.7.1 to detect sequence homology with a *T. vivax VSG* database. This information is added to a regularly updated presence/absence binary matrix of *T. vivax* genomes and applied to a Euclidean distance-based clustering analysis. The results are presented as a heat map and dendrogram, putting the sample in the context of the available *T. vivax* genomes.

For *T. vivax* transcriptomic analyses (tVAP), VAPPER works similarly to *T. congolense* but using a VSG database rather than protein motifs. Because sequencing depth is generally not great enough to exhaustively detect all VSGs in a single sample, and to accommodate the substantial number of strain-specific VSGs, using the raw VSG database is not the most tractable approach. Therefore, to make the *T. vivax* transcriptomic analysis exhaustive and consistent with the *T. congolense* tVAP, we defined 174 phylotypes that combine VSGs with a sequence identity score of ≥70%. In the presence of low genome coverage, it is essential to adopt a phylotype-based system rather than dealing with individual genes.

In its Linux version, VAPPER can process multiple samples concurrently, providing that the input files are compiled in a single directory. Results are shown for all samples simultaneously, allowing direct comparison of variant antigen profiles across multiple isolates, conditions, or replicates. The tabular output can be incorporated in downstream statistical analysis, whilst the graphical outputs provide figures for the visualization of antigen repertoire variability.

### Linux package installation

To facilitate use, the installation of VAPPER and its dependencies is automated. Upon first download of the software, a single script will ensure that the system has all the required dependencies and install them in a local directory if necessary. In naive environments and for users without administrator rights to install the necessary libraries, a Python virtual environment can be set upon each new session. A step-by-step guide for the installation and use of VAPPER can be found in Supplementary File 1.

### The galaxy tool

**Figure 2:**
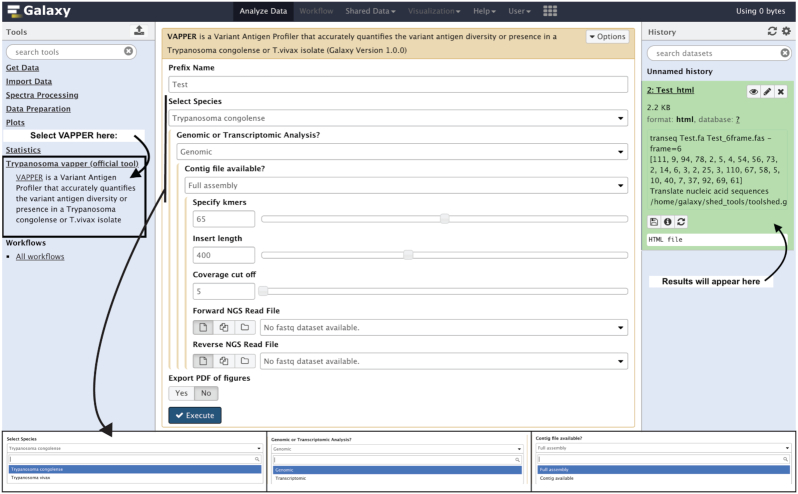
Screenshot of VAPPER on the Galaxy interface. This interface is available after installation of VAPPER from the Galaxy ToolShed [20] into a local Galaxy server. In this case, VAPPER was installed on the University of Liverpool Galaxy server. The blue panel on the right shows how to search and select VAPPER after installation. The white panel at the centre shows the options available for the user, including the prefix name of the sample to appear on the output figures, the species, and the type of input data. If any genomic pipeline is selected, further options for genome assembly parameters are available. Finally, the user can choose whether to get the graphs in PDF format (default is PNG only).

VAPPER is available for installation in local Galaxy servers from the Galaxy ToolShed [32]. The purpose of the incorporation of VAPPER in Galaxy local servers is to provide a simple front-end component for inexperienced users (Fig. 2). Results can be visualised directly in Galaxy or can be downloaded as a compressed folder containing an HTML file with combined results, individual PNG and PDF files of the heat maps, PCA plots, and bar charts produced, and the CSV files containing the raw values of phylotype proportions and deviation from the mean. A step-by-step guide for the installation of VAPPER on a Galaxy local server can be found in Supplementary File 1.

### Benchmarking

**Figure 3:**
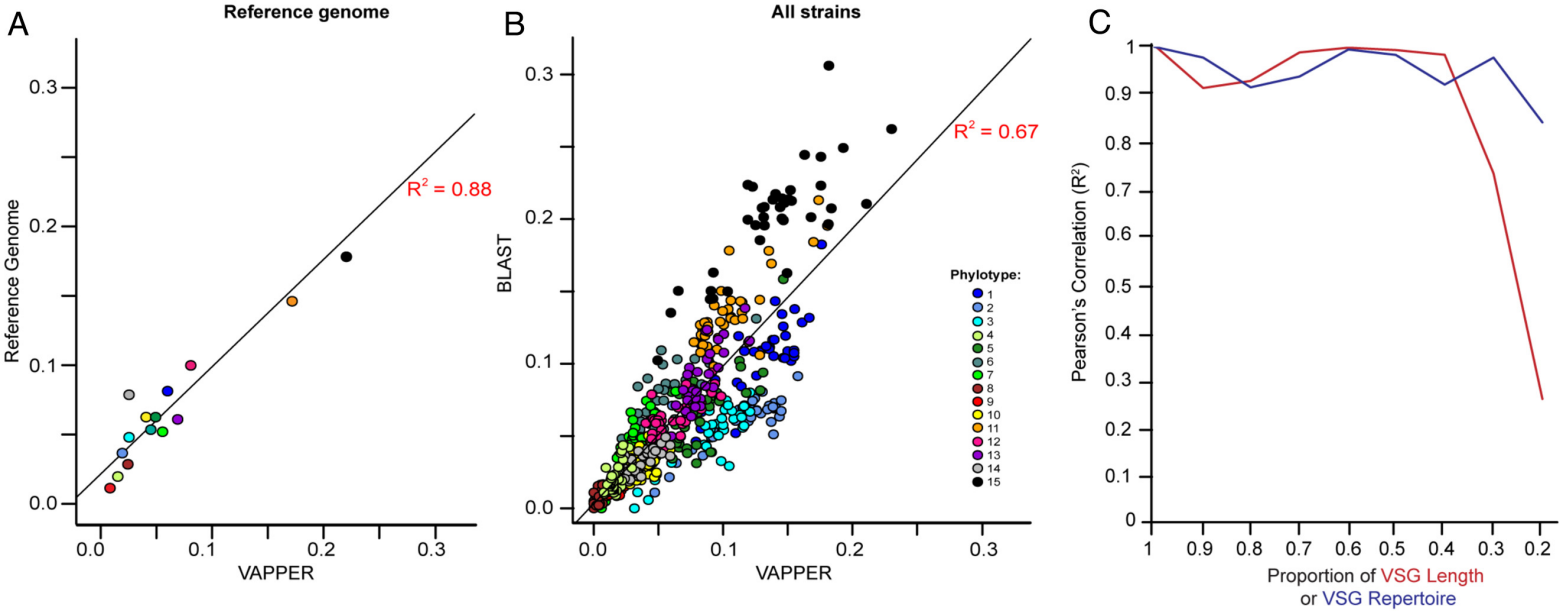
VAPPER performance (*T. congolense* genomic pipeline). (A) Correlation of phylotype frequencies produced by VAPPER and those manually curated in the *T. congolense* IL3000 reference genome sequence [7]. Pearson's product moment correlation statistics: *R*^2^ = 0.88, *t*(13) = 9.7321, *P*-value < 0.001. (B) Correlation of phylotype frequencies produced by VAPPER and BLAST-based [33] phylotype frequencies in a panel of 41 *T. congolense* strains. Pearson's product moment correlation: *R*^2^ = 0.67, *t*(566) = 34.39*P-value* < 0.001. (C) VAPPER accuracy in fragmented (red) or incomplete (blue) genomes. Line graphs show correlations of the expected antigen profiles of a known set of VSG sequences from the IL3000 genome sequence with antigen profiles produced from fragmented VSGs or incomplete VSG repertoires. Fragmentation and genome incompletion were simulated from random sampling. Gene fragmentation was calculated as a proportion of the mean length of the original VSG sequences (mean ± σ = 1,163 ± 129 nucleotides). Figure adapted from Silva Pereira et al. [18].

The performance of the *T. congolense* gVAP pipeline was compared to the manually annotated VAP of the IL3000 reference genome (Fig. 3A) and to the BLAST-based VAPs of 41 isolates (Fig. 3B) [18]. There is a very good correlation between profiles produced by VAPPER and the known IL3000 VAP (*R*^2^ = 0.88, *t*_(13)_= 9.73, *P*-value < 0.001) and a good correlation with the BLAST-based method (*R*^2^ = 0.67, Pearson's product moment correlation, *t*_(566) =_ 34.39, *P*-value < 0.001). Minor differences were further investigated and found to be due to BLAST's difficulty in either analysing small contigs or quantifying multiple VSGs in the same contig sequence. Therefore, in general, more VSGs were recovered with VAPPER than with BLAST (mean ± σ = 721 ± 277 vs 669 ± 292, paired *t*-test, *P*-value = 0.005). A further strength of VAPPER is the ability to deal with poor, fragmented genome assemblies. As described in our previous article [18], when a single *VSG* gene is located in 2 distinct contig fragments, BLAST counts them incorrectly as separate genes, whereas VAPPER will not because the diagnostic motif is only present once. Therefore, we can now accurately calculate antigen profiles from incomplete genome assemblies (up to 30%), and with a *VSG* fragmentation level up to 40% of the original gene length (223 nucleotides) (Fig. 3C).

#### Validation by example

##### *T. congolense* gVAP

**Figure 4:**
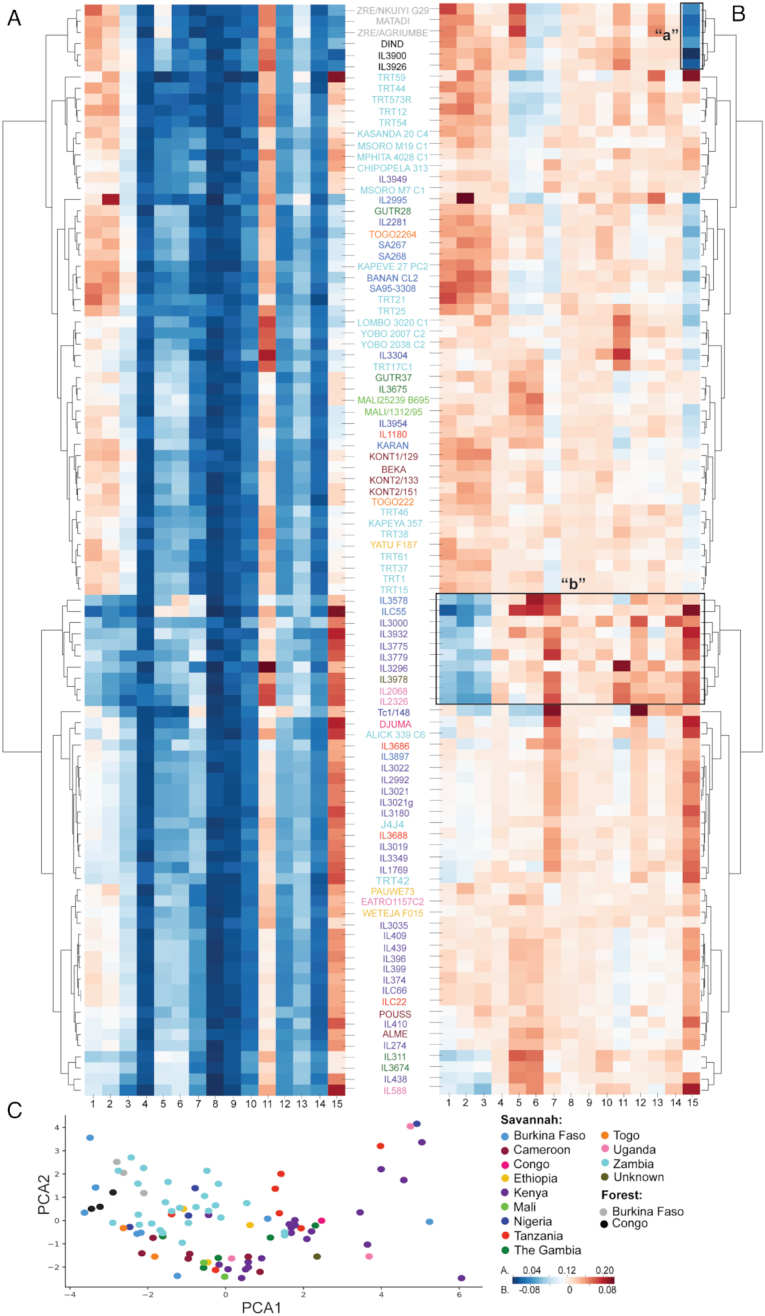
VAPPER output for *T. congolense* genomic pipeline. (A) Heat map and corresponding dendrogram showing the variant antigen profiles (VAP) of the current genomic database expressed as phylotype frequencies [18, 26]. (B) Heat map and corresponding dendrogram showing the VAPs of the current genomic database expressed as deviation from the mean phylotype frequency [18, 26]. Labels “a” and “b” are referred to in the text. (C) PCA plot representing variation in *VSG* repertoire across the *T. congolense* genomic database [18, 26] (N = 97).

We have used the VAPPER to analyse the genomic repertoire of 98 *T. congolense* samples of savannah and forest subtypes, collected from 12 countries across Africa, and previously described by us [18] and others [26]. In Fig. 4, 2 heat maps and corresponding dendrograms show how the VSG repertoires of each strain relate to each other. On the left, the heat map represents phylotype proportion, i.e., how many genes a specific phylotype contains in the context of the complete *VSG* repertoire for a given strain (Fig. 4A). This heat map shows that P4, 8, 9, 10, and 14 have few genes in all strains, whereas other phylotypes (e.g., P1, 2, 15) are more variable, being quite abundant in some strains and rare in others. The heat map on the right shows phylotype deviation from the mean (Fig. 4B), which is calculated as the difference between the phylotype proportion shown in panel A and the arithmetic mean of phylotype proportions. The latter is calculated from the current database; thus, it will change as new samples are added.

The phylotype proportion variation patterns are perhaps better detected in the normalized heat map (Fig. 4B). For example, it is possible to detect a signature of underrepresented P15 characteristic of all forest-subtype samples (denoted by “a”), abundant P15 in all Kenyan isolates (in purple), as well as a distinct pattern characteristic of strains IL3578 to IL2326, characterized by the combination of low P1–3 and high P7 (denoted by “b”). The latter does not seem to be related to geography because it encompasses isolates from Kenya, Uganda, and Burkina Faso. The PCA plot further indicates that VSG repertoires and geography are only weakly correlated (Fig. 4C), which agrees with our previous observation that *T. congolense* VSG repertoires do not mimic either population structure or geography [18].

##### *T. congolense* tVAPs

**Figure 5:**
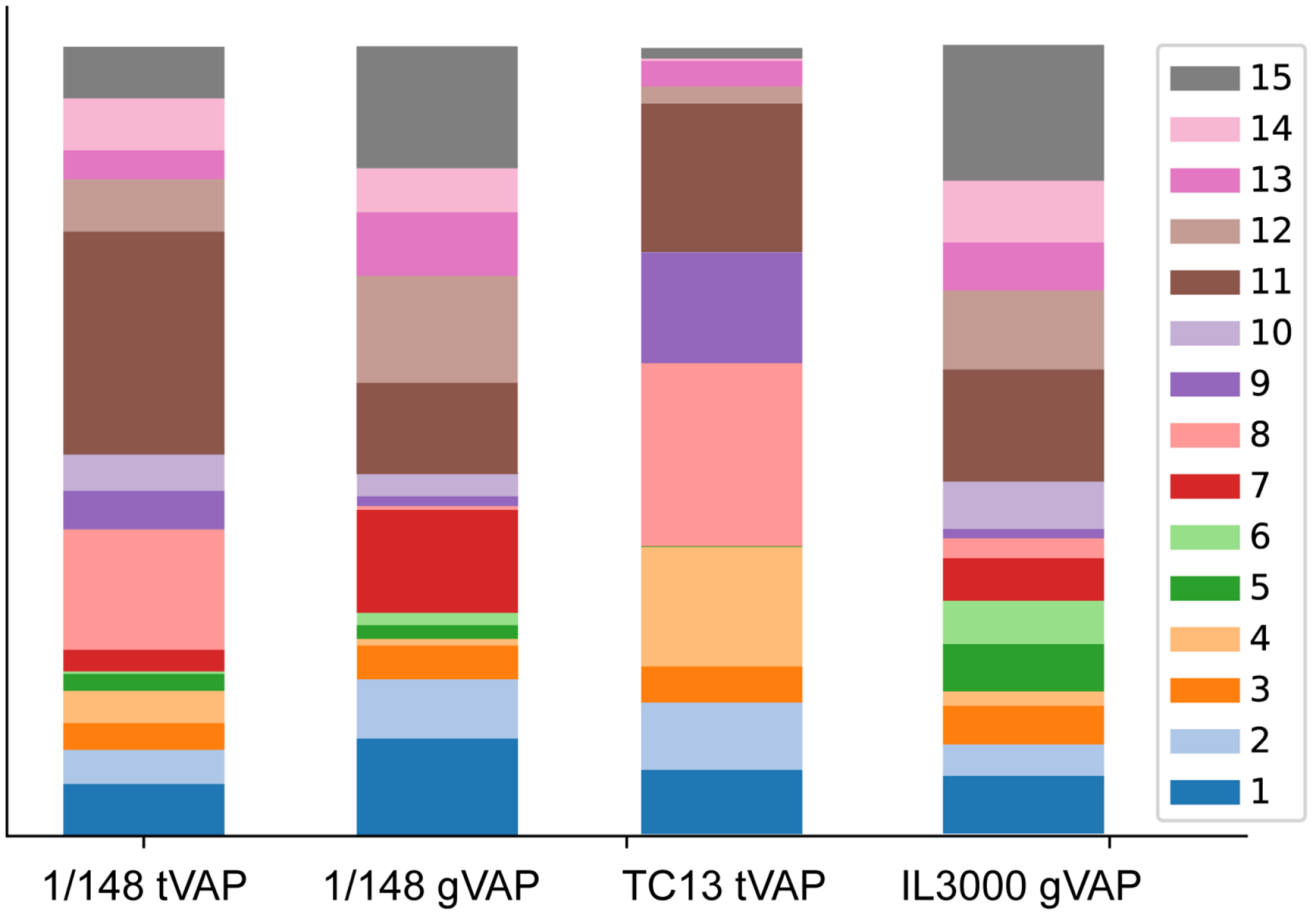
VAPPER output for *T. congolense* transcriptomic pipeline. Stacked bar charts showing expressed variant antigen profiles (VAPs) of metacyclic-stage *T. congolense* from strain 1/148 [18] and TC13 [34] compared to the genomic repertoires of the same strain (1/148) or a closely related one (IL3000) [29]. Phylotypes are colour-coded according to key. Size of each stack represents proportion of the phylotype relative to the total repertoire of expressed *VSGs*.

We have used VAPPER to analyse the expressed VSG repertoire of the metacyclic (infective) life stage of *T. congolense*. For that, we have produced a tVAP for the strain TC13, whose transcriptome was published by Awuoche et al. [34]. We have compared the metacyclic tVAP of this strain with the 1/148 strain (MBOI/NG/60/1-148) that we have previously described [30]. Furthermore, we have compared them to the genomic *VSG* repertoires of the same strain, or a related one (Fig. 5). Because we do not have a genome sequence for the TC13 isolate, we compared it to IL3000, which was isolated in the same region (Transmara, Kenya) [35].

When we compare the gVAPs of 1/148 and IL3000, we see that they are distinct, and so are the tVAPs (e.g., P4 is more represented in TC13, whereas P10 is more represented in 1/148 than in TC13). However, P8 is overrepresented in both isolates compared to the genomic repertoires (Fig. 5). This agrees with our previous observation that the pattern of metacyclic *VSG* expression is significantly different from the genome repertoires, and that the metacyclic *VSG* repertoire is particularly enriched for P8 genes [18]. With the analysis of the TC13 transcriptome, we can now add that this enrichment does not seem to be strain-specific, but rather equally applicable to *T. congolense* strains of distinct backgrounds.

##### *T. vivax* gVAP

**Figure 6:**
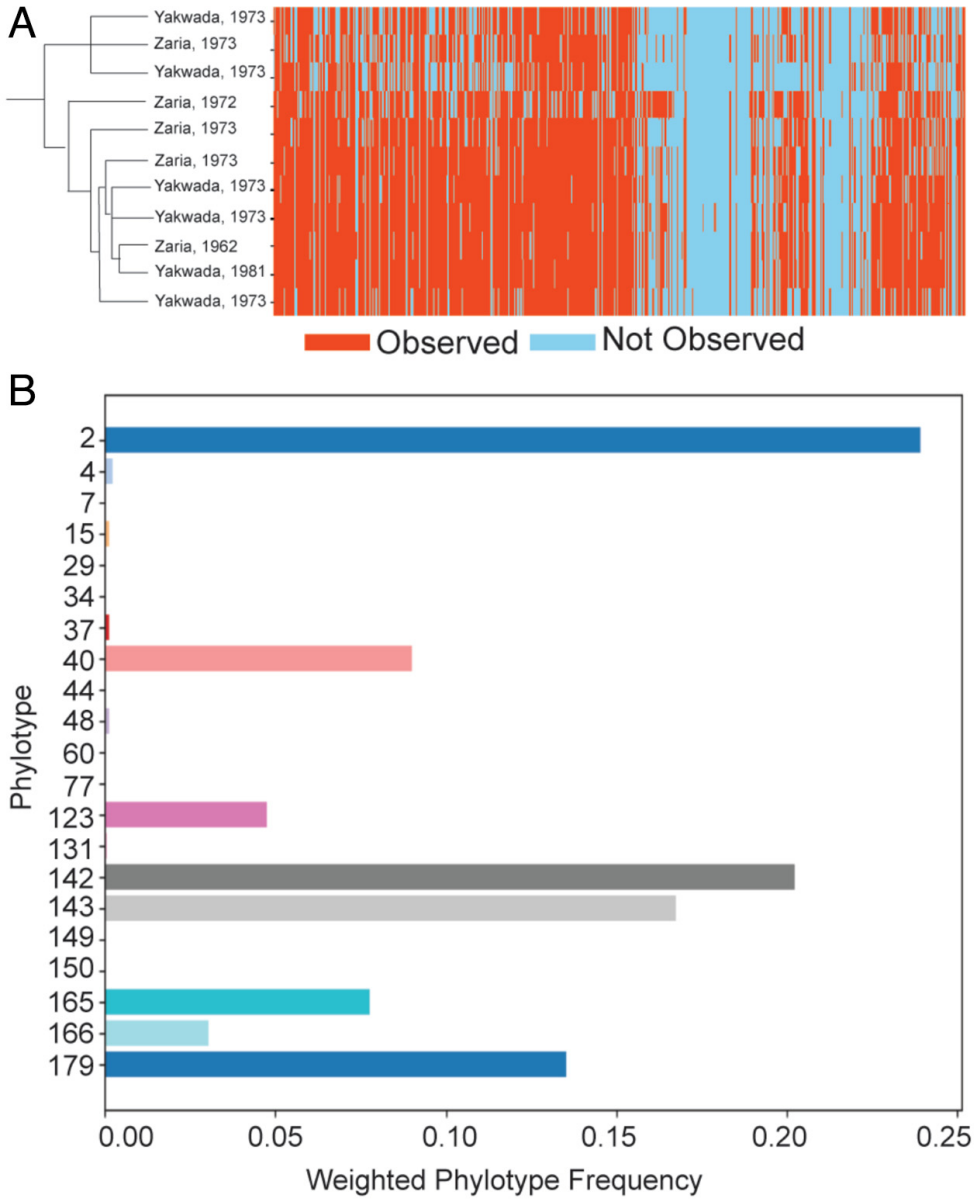
VAPPER output for *T. vivax* genomic and transcriptomic pipelines. (A) Heat map and corresponding dendrogram showing *T. vivax* variant antigen profiles (VAPs) of 11 Nigerian strains. (B) Expressed VAP of *T. vivax* IL1392 bloodstream form previously published by Jackson et al. [36], shown as a bar chart.

The *T. vivax* gVAP shows the VAPs in the context of a *T. vivax* genome database. As proof of concept, we have produced VAPs for 11 isolates collected across Nigeria. The dendrogram represents the relationships between the multiple samples, whereas the heat map shows whether *VSG* genes are present or absent in each of them (Fig. 6A). Overall, these profiles show high reproducibility across samples, as would be expected for isolates of similar geographical location. Yet, the profiles also reveal some differences amongst isolates, suggesting strain-specific variation and highlighting the potential epidemiological value of particular *VSG* genes.

##### *T. vivax* tVAP

As proof of concept, we have used tVAP to obtain the expressed VAP of the previously published strain IL1392 [36] (Fig. 6B). We observe 21 phylotypes being expressed, of which 8 have a substantial weight. P2 is the most abundant phylotype, followed by P142 and P143. These profiles can be used to compare VSG repertoires and identify phylotype patterns that may be epidemiologically relevant, perhaps contributing to the considerable phenotypic variation observed in *T. vivax* AAT.

We understand that the expansion of VAPPER to the widely studied, human-infective species *T. brucei* will have great value to the community. Current *T. brucei VSG* analyses are extensive and thorough, but strain-specific because the extremely dynamic, highly recombinant *VSG* repertoire is a challenge for profiling approaches. This task will be possible in the future but will definitely require a novel methodology. Attempts to profile *VSG* genes based on amino acid signatures such as the one presented here for *T. congolense* will likely fail owing to the extreme degree of mosaicism [37] and the ability to convert genes between very diverse donor regions [38]. If a minimal *VSG* recombination unit can be determined, alternative systematics may resort to mosaic frequencies, particularly because mosaics formed from the same set of genes can have higher nucleotide identity between themselves than their precursors. In fact, alternative antigenic profiling methods already exist for some organisms. For example, profiling of *P. falciparum var* gene diversity was achieved through a population genomic framework [39] targeting variation in the Duffy binding-like α (DBLα) motif, a ubiquitous 500-nucleotide fragment marker. Yet, for other pathogens with variant multi-copy gene families, such as *Trypanosoma cruzi*, antigen profiling has not yet been done. For these, species- and gene family–specific motif-based approaches, such as the one presented here for *T. congolense*, may offer a tractable solution.

## Conclusion

VAPPER is the first tool for the systematic analysis of *VSG* gene and expression diversity across strains and during infections. It establishes a practical approach for measuring antigenic diversity in these important pathogens based on universal protein motifs and/or gene mapping. VAPPER allows us to identify and characterize differences in antigenic repertoires between strains, hosts, and conditions, which may be the starting point to build a real understanding of the association between parasite genotypes and outcomes of AAT.

## Availability of source code and requirements

Project name: VAPPER—High-throughput Variant Antigen Profiling in African Trypanosomes

Project home page: https://github.com/PGB-LIV/VAPPER

Operating system: Platform independent

Programming language: Python

Installation requirements: Velvet 1.2.10, HMMER 3.1b2, Bowtie 2 2.2.6, SAMtools 1.6, Cufflinks 2.2.1, BLAST 2.7.1, EMBOSS

License: Apache v.2.0

RRID:SCR_016993

## Availability of supporting data and materials

Snapshots of our code and other data further supporting this work are available in the GigaScience repository, GigaDB [40].

## Additional files

SupplementaryFile1_VAPPER_User_Guide.docx

giz091_GIGA-D-18-00480_Original_Submission

giz091_GIGA-D-18-00480_Revision_1

giz091_Response_to_Reviewer_Comments_Original_Submission

giz091_Reviewer_1_Report_Original_Submission Igor Cestari -- 1/3/2019 Reviewed

giz091_Reviewer_1_Report_Revision_1 Igor Cestari -- 6/26/2019 Reviewed

giz091_Reviewer_2_Report_Original_Submission Sebastian Hutchinson -- 2/4/2019 Reviewed

giz091_Supplemental_File

## Abbreviations

AAT: animal African trypanosomiasis; BLAST: Basic Local Alignment Search Tool; FPKM: fragments per kilobase million; gVAP: genomic variant antigen profile; HMM: hidden Markov model; PCA: principal component analysis; tVAP: transcriptomic variant antigen profile; VAP: variant antigen profile; VAPPER; Variant Antigen Profiler; VSG: variant surface glycoprotein.

## Competing interests

The authors declare that they have no competing interests.

## Funding

This work was supported by a Grand Challenges (Round 11) award from the Bill and Melinda Gates Foundation, a BBSRC New investigator Award (BB/M022811/1), and the Technology Directorate of the University of Liverpool to A.P.J.

## Authors' contributions

S.S.P. wrote the original code in Perl and tested the software. J.H. and A.R.J. wrote the final code in Python. S.S.P. and A.P.J. conceptualized the software and wrote the manuscript. All authors contributed to and approved the final manuscript.
