# Supplementary material for: VAPPER: High-throughput variant antigen profiling in African trypanosomes of livestock
Source: Gigascience. 2019 Aug 29;8(9):giz091. doi: 10.1093/gigascience/giz091 (PMC6735694; doi:10.1093/gigascience/giz091)
Supplement: giz091_GIGA-D-18-00480_Revision_1 [file giz091_giga-d-18-00480_revision_1.pdf]

GigaScience

VAPPER: High-throughput Variant Antigen Profiling in African trypanosomes of Livestock

--Manuscript Draft--

Manuscript Number:	GIGA-D-18-00480R1
Full Title:	VAPPER: High-throughput Variant Antigen Profiling in African trypanosomes of Livestock
Article Type:	Technical Note
Funding Information:	Biotechnology and Biological Sciences Research Council (BB/M022811/1) — Dr Andrew P. Jackson<br>Bill and Melinda Gates Foundation (US) (Grand Challenges (Round 11)) — Dr Andrew P. Jackson<br>Technology Directorate of the University of Liverpool — Dr Andrew P. Jackson

Abstract:

Background

Analysing variant antigen gene families on a population scale is a difficult challenge for conventional methods of read mapping and variant calling due to the great variability in sequence, copy number and genomic loci. In African trypanosomes, hemoparasites of humans and animals, this is complicated by variant antigen repertoires containing hundreds of genes subject to various degrees of sequence recombination.

Findings

We introduce Variant Antigen Profiler (VAPPER), a tool that allows automated analysis of the variant surface glycoprotein repertoires of the most prevalent livestock African trypanosomes. VAPPER produces variant antigen profiles for any isolate of the veterinary pathogens Trypanosoma congolense and Trypanosoma vivax from genomic and transcriptomic sequencing data and delivers publication-ready figures that show how the queried isolate compares with a database of existing strains. VAPPER is implemented in Python. It can be installed to a local Galaxy instance from the ToolShed (https://toolshed.g2.bx.psu.edu/) or locally on a Linux platform via the command line (https://github.com/PGB-LIV/VAPPER). The documentation, requirements, examples, and test data are provided in the Github repository.

Conclusion

By establishing two different, yet comparable methodologies, our approach is the first to allow large-scale analysis of African trypanosome variant antigens, large multi-copy gene families that are otherwise refractory to high-throughput analysis.

Corresponding Author:	Sara Silva Pereira<br>Instituto de Medicina Molecular "João Lobo Antunes"<br>Lisboa, PORTUGAL
Corresponding Author Secondary Information:	
Corresponding Author's Institution:	Instituto de Medicina Molecular "João Lobo Antunes"
Corresponding Author's Secondary Institution:	
First Author:	Sara Silva Pereira
First Author Secondary Information:	
Order of Authors:	Sara Silva Pereira
	John Heap
	Andrew R. Jones

Powered by Editorial Manager® and ProduXion Manager® from Aries Systems Corporation

	Andrew P. Jackson
**Order of Authors Secondary Information:**	
**Response to Reviewers:**	17th June 2019<br><br>Dear Editor,<br><br>Re:. GIGA-D-18-00480 - VAPPER: High-throughput Variant Antigen Profiling in African trypanosomes of Livestock<br><br>We are grateful to you for the chance to resubmit our manuscript to GigaScience. We note that both reviewers are content that our main observations and interpretation. The comments from each reviewer were most valuable. Particularly, by addressing the concerns of Reviewer 1, we have improved the manuscript and increased the usability of the tool by providing an additional pipeline for the analysis of Trypanosoma vivax transcriptomic data (with an example shown in Figure 6B) and more description of installation and usage procedures. The latter has been addressed by carefully preparing a step-by-step user guide. Please note that we have changed Figure 6A, which previously showed a collection of isolates from across Africa, to show a more densely sampled set of same Nigerian location over multiple time points. We think this highlights the reproducibility of VAPPER in the best possible way, and shows how VAPPER can be used to provide epidemiological data within an authentic disease setting. Furthermore, and as suggested, we have included discussion of the scope of the tool and the importance of variant antigen profiles. Our responses to each specific comment are given below.<br><br><br>Responses to Reviewers' Comments:<br><br>Reviewer #1:<br>This is a useful tool for analysis of VSGs in trypanosomes of veterinary importance. The tool will support the analysis of VSG gene families which is challenging due to their abundance, mutation and/or recombination rates. A concern is VAPPER limitation to trypanosomes of veterinary importance, and thus the preliminary character of this work. It would be relevant to have it available to other trypanosomes, e.g. T. brucei brucei. Also, T. brucei that infect humans (e.g. T. brucei gambiense or rhodesiense), especially given the number of transcriptomes and genomes available for these parasites and the ability to track changes in VSG repertoire during infections with sequencing technologies. Also, it seems preliminary that the authors did not include validation of their tool with transcriptome of T. vivax, which is due to the lack of enough (as indicated by authors) transcriptome data. This work deserves to be published; however, it would be advantageous to include the option for analysis of variant antigens of other pathogens, at least the closest related T. brucei and additional validation to be complete.<br><br>Specific comments:<br>The limitation of the tool for two pathogens is discouraging, and I am astonished that the software did not include analysis of VSGs from T. brucei spp. which causes animal and human African trypanosomiasis. The authors also did not justify why they are not including an analysis of T. brucei spp. VSG genes since there is potential scientific interest from those working in the T. brucei field. Also, the implementation of VAPPER for analysis of other large antigen gene families such as those in T. cruzi, Leishmania, and Plasmodium etc was not included or discussed.<br><br>R.<br>We would like to thank reviewer 1 for his comments. We have carefully considered them and will address them individually below.<br><br>My suggestions are:<br>1)Authors should address why VAPPER does not analyze multigene families of other pathogens, and it should add the option to analyze (at least) T. brucei VSGs. The title of the manuscript and its introduction is suggestive of a broad utility of the tool for African trypanosomes, and not T. vivax and T. congolense only. This only became apparent much later in the text.

Powered by Editorial Manager® and ProduXion Manager® from Aries Systems Corporation

R.
Please be assured that including a T. brucei Variant Antigen Profile within VAPPER is a priority for us. Its omission is certainly not an oversight. The problem is that antigenic diversity in T. brucei is much more complex than T. congolense and T. vivax, and the computational solution to comparing any set of T. brucei strains is not yet available. Nevertheless, since T. brucei typically contributes less than 5% of cattle infections and there are many experiments now being devoted to T. congolense and T. vivax, we feel that there is worth in releasing the existing tools.

VSGs are refractory to conventional techniques of gene mapping because they recombine more than a normal gene. In T. congolense, this recombination is restricted to genes of the same cohort (or phylotype); therefore, we could design phylotype-specific protein motifs that are universal to any T. congolense strain. In T. vivax, the VSG recombination rate is generally low, which allows us to find the same gene across species; therefore, we could design a strategy based on clusters of gene orthologs. This is not the case in T. brucei; the VSG repertoire in this species is extremely dynamic, and virtually every VSG gene is a mosaic, likely to be found in other strains as a number of dispersed fragments around the genome. Very few T. brucei VSG are routinely found as orthologs across diverse strains.

So, while the reviewer correctly mentions that there are plenty of T. brucei genomes and transcriptomes available and that we can track changes in VSG repertoire during infections with current sequencing technologies, there are considerable problems to comparing VSG repertoire across multiple T. brucei strains. This is the main goal of VAPPER, producing a VAP for any isolate(s). Every T. brucei VSG analysis done to date has been developed on single strains, usually the same lab strain or its descendants, and VSG nomenclature for most parts has been study-specific. The picture is very different when we compare different clinical strains; then there is very little orthology and certainly mapping VSG reads from a clinical isolate onto a reference genome would make little sense. This dynamism and low sequence orthology between isolates mean that developing a VSG systematics universal to all T. brucei strains is an enormous challenge. We have included a paragraph discussing this and proposing alternative methodologies that may work best for this pathogen (ll. 279-289). We also address established variant antigen profiling methods in other pathogens (e.g. P. falciparum) and how a similar motif-based, but gene family-specific methodology could be developed for T. cruzi (ll. 289-297).

For clarity regarding the scope of VAPPER, we have changed the title, the abstract, and introduction to reiterate that VAPPER is a tool for the most prevalent livestock trypanosomes, T. congolense and T. vivax.

2)Validation of tVAP for T. vivax was not done due to the lack of transcriptomic data. How much data is needed for validation of this part of the tool? Because tVAP analysis for T. vivax uses a different pipeline than that of T. congolense, the validation of T. congolense does not guarantee that VAPPER would perform well for T. vivax tVAP. Can the authors generate the data to test the tool? This is another limitation of the tool which indicates that the work is still preliminary, and the authors should consider some additional effort to conclude this work prior publication. I do encourage the authors to do so, as VAPPER has the potential to be of interest in the parasitology community.

R.
We have developed (ll. 159-167) and validated (ll. 271-277) tVAP for T. vivax and included it in the revised VAPPER package.

Minor comments:
1) The author should include in the introduction why is relevant to study the variant antigen profile of a pathogen and indicate examples. That VSGs are essential for immune evasion is clear, but it is not so clear the advantages (or when it would be necessary) to study the whole repertoire of VSGs and separate them in distinct clades

Powered by Editorial Manager® and ProduXion Manager® from Aries Systems Corporation

	(or groups). One possibility is that the analysis of variant antigen repertoires over an infection, which might provide insights into recombination mechanisms or other processes. Although some examples are included in the Results, it would be good to have examples of potential applications in the Introduction.<br><br>R.<br>We have introduced this in the introduction (ll. 98-105).<br><br><br>2) I found it difficult to install the tool using galaxy, and I did not find much instruction on how to proceed with this in the manuscript. I do have some experience in bioinformatics with analysis of RNAseq, proteome or other tools using R. However, galaxy is new for me (a molecular biologist with some knowledge in bioinformatics) and having some step by step description on how to install VAPPER would have helped. The lack of instructions may discourage the use of the software by others. Since the authors indicate that deposition of the tool through galaxy toolshed was to help less experienced users, it did not help me much. Hence, some additional description in the manuscript or indication of a step by step protocol (e.g. as supplementary material) on how to access and install the VAPPER would be useful, including computer requirements etc.<br><br>R.<br>The galaxy toolshed is a shared repository with rules and procedures that surpass us as the authors. However, we have compiled a detailed protocol as supplementary file 1, where we provide a step-by-step description on how to install VAPPER both locally and using Galaxy. This user guide also includes a description of the program usage, commands examples, and instructions to test the installation using test data provided in the Github directory.<br><br>Reviewer #2: This paper reports a pipeline, VAPPER, for analysing VSG antigen profiles present in the genomes and transcriptomes of the important animal pathogens Trypanosoma congolense and T. vivax. VAPPER is well documented and executed, was easy to install and test for this review (on Mac OSX) and performed as advertised.<br><br>(Very) minor points:<br>line 73: Typo - missed space<br>line 114-115: Sentence needs rewording (missing 'are'?)<br><br>R.<br>We would like to thank reviewer 2 and confirm that these minor points have been addressed.
**Additional Information:**	
**Question**	**Response**
Are you submitting this manuscript to a special series or article collection?	No
**Experimental design and statistics**<br><br>Full details of the experimental design and statistical methods used should be given in the Methods section, as detailed in our Minimum Standards Reporting Checklist. Information essential to interpreting the data presented should be made available in the figure legends.	Yes

Have you included all the information requested in your manuscript?	
**Resources**<br><br>A description of all resources used, including antibodies, cell lines, animals and software tools, with enough information to allow them to be uniquely identified, should be included in the Methods section. Authors are strongly encouraged to cite Research Resource Identifiers (RRIDs) for antibodies, model organisms and tools, where possible.<br><br>Have you included the information requested as detailed in our Minimum Standards Reporting Checklist?	Yes
**Availability of data and materials**<br><br>All datasets and code on which the conclusions of the paper rely must be either included in your submission or deposited in publicly available repositories (where available and ethically appropriate), referencing such data using a unique identifier in the references and in the “Availability of Data and Materials” section of your manuscript.<br><br>Have you have met the above requirement as detailed in our Minimum Standards Reporting Checklist?	Yes

Manuscript with DOI

Click here to access/download;Manuscript;VAPPER-Revised W DOI.docx

Click here to view linked References

VAPPER: High-throughput Variant Antigen Profiling in African trypanosomes of Livestock

Sara Silva Pereira[1¶*], John Heap[2], Andrew R. Jones[3], Andrew P. Jackson[1*]

1.

Department of Infection Biology

Institute of Infection and Global Health

University of Liverpool

Liverpool Science Park Ic2

146 Brownlow Hill

Liverpool L3 5RF

United Kingdom

2.

Computational Biology Facility

University of Liverpool

Liverpool L69 7ZB

United Kingdom

3.

Institute of Integrative Biology

University of Liverpool

Liverpool L69 7ZB

United Kingdom

[¶] Current Address:

Instituto de Medicina Molecular - João Lobo Antunes, Faculdade de Medicina, Universidade de Lisboa, Lisbon, Portugal

*Corresponding authors
Email: ssilvapereira@medicina.ulisboa.pt
Email: a.p.jackson@liverpool.ac.uk

Abstract

Background: Analysing variant antigen gene families on a population scale is a difficult challenge for conventional methods of read mapping and variant calling due to the great variability in sequence, copy number and genomic loci. In African trypanosomes, hemoparasites of humans and animals, this is complicated by variant antigen repertoires containing hundreds of genes subject to various degrees of sequence recombination. **Findings:** We introduce Variant Antigen Profiler (VAPPER), a tool that allows automated analysis of the variant surface glycoprotein repertoires of the most prevalent livestock African trypanosomes. VAPPER produces variant antigen profiles for any isolate of the veterinary pathogens *Trypanosoma congolense* and *Trypanosoma vivax* from genomic and transcriptomic sequencing data and delivers publication-ready figures that show how the queried isolate compares with a database of existing strains. VAPPER is implemented in Python. It can be installed to a local Galaxy instance from the ToolShed (https://toolshed.g2.bx.psu.edu/) or locally on a Linux platform via the command line (https://github.com/PGB-LIV/VAPPER). The documentation, requirements, examples, and test data are provided in the Github repository. **Conclusion**: By establishing two different, yet comparable methodologies, our approach is the first to allow large-scale analysis of African trypanosome variant antigens, large multi-copy gene families that are otherwise refractory to high-throughput analysis.

Keywords: VAPPER; variant antigen profiling; African trypanosomes; variant surface glycoproteins

Background

Advances in next-generation sequencing have enabled researchers to produce high-throughput genomic data for diverse pathogens. However, analysing multi-copy, contingency gene families remains challenging due to their abundance, high mutation and recombination rates, and unstable gene loci [1]. Yet, these gene families are often involved in many processes of pathogenesis, including antigenic variation, virulence, host use, and immune modulation in a multitude of pathogens [2–4]. A prime example of a crucial gene family lacking the necessary analytic tools for high-throughput analysis is the Variant Surface Glycoprotein (*VSG*) superfamily in African trypanosomes [5].

African trypanosomes are extracellular hemoparasites that cause human sleeping sickness and animal African trypanosomiasis (AAT). Their genomes contain up to 2500 *VSG* genes [6,7] dispersed through specialized, hemizygous chromosomal regions called subtelomeres, smaller chromosomes, and less frequently in the core of megabase-sized diploid chromosomes. The *VSG* genes encode variant surface glycoproteins, GPI-anchored proteins that coat the entire surface of the parasite in the bloodstream of the mammal host, which function mostly in antigenic variation and immune-modulation [8]. Sporadically, specific *VSG* genes have been shown to evolve other functions, not related to antigenic variation, such as conferring human infectivity to *T. brucei gambiense* (*TgsGP* gene) [9,10] and *T. brucei rhodesiense* (*SRA* gene) [11,12], resistance to the drug suramin (*VSG*^{sur} gene) [13], and mediating the transport of transferrin (*TfR* genes) [7,14].

As they are key players in host-trypanosome interaction, understanding *VSG* diversity and its impact in pathology, disease phenotype and virulence is of foremost importance in trypanosome research [4]. However, the *VSG* repertoire cannot be accurately analysed using conventional approaches of read mapping and variant calling. Attempts to bypass this challenge have resulted in alternative approaches using manually-curated *VSG* gene databases for specific *T. brucei* strains [6,15–17], but to the best of our knowledge there is no automated tool for the systematic analysis of *VSG* from any trypanosome genome. Thus, we have developed Variant Antigen Profiler (VAPPER), a tool that examines *VSG* repertoires in DNA/RNA sequence data of the main livestock trypanosomes, *Trypanosoma congolense* and *T. vivax*, and quantifies antigenic diversity. This results in a variant antigen profile (VAP) that can be compared between isolates, locations, and experimental conditions [18].

Studying variant antigen profiles may reveal important aspects of the host-pathogen interaction. For example, we have recently shown that *T. congolense* phylotype 8 transcripts are abundant in metacyclic parasites, and that this abundance is attributed to the phylotype in its entirety rather than a specific gene [18]. Similarly, in *P. falciparum*, Group A *var* genes as a whole, and not individual genes, have been linked to severe disease [19]. Therefore, for some purposes, studying variant antigen profiles can be more informative than individual gene analysis. In this paper we briefly present how VAPPER can be used to further our knowledge of antigenic diversity and variation.

Findings

The service

VAPPER is primarily intended for producing and comparing VAPs of livestock trypanosomes, without the need for complex bioinformatic processes. It is available online through the Galaxy ToolShed [20] for a local Galaxy server [21], and as a Linux

package for local installation. The program has four pipelines, specific for each organism (*T. congolense* or *T. vivax*) and input data type (genome or transcriptome). VAPPER requires quality-filtered, trimmed, paired sequencing reads in FASTQ format [22] or assembled contigs in FASTA format [23]. Results are presented in tables of frequencies, heatmaps, and Principal Component Analysis (PCA) plots, visualized as HTML files or exported to PDF or PNG format. A typical workflow is shown in Fig. 1.

For *T. congolense* genomic VAPs (gVAP), VAPPER starts with genome assembly of raw, short reads using Velvet 1.2.10 [24]. Assembled contigs are screened for pre-defined protein motifs described by a hidden Markov model using HMMER 3.1b2 [25] after 6-frame translation. A detailed description of the universal protein motifs and their biological significance is presented in a recent manuscript [18], but, in summary, each protein motif or motif combination is diagnostic of a specific phylotype [18]; therefore, phylotype frequency can be calculated from the HMMER output. The proportions of each phylotype represent the gVAP and are recorded in a table of frequencies. The gVAP produced is also placed in the context of a *T. congolense* genome database supplied with VAPPER (N=97, [18,26]), which is regularly updated. This is achieved through a Euclidean distance-based clustering analysis. Results are presented as two heatmaps with corresponding dendrograms, one showing phylotype frequency, and the other showing frequency deviation from the population mean. They are also shown as a PCA plot and a table of frequencies.

For *T. congolense* transcriptomic analyses (tVAP), VAPPER performs read mapping using Bowtie 2 2.2.6 [27], reference-based transcript assembly and abundance calculation using Cufflinks 2.2.1 [28], and *VSG* transcript screening and phylotype assigning as described for gVAP. The proportions of each phylotype are then adjusted for transcript abundance based on the Cufflinks output. The tVAP is presented as a weighted bar chart and compared to the gVAP of the reference. Ideally, the user would

provide their own reference genome for the mapping step. As that is not always possible, especially for field isolate analysis, we provide two reference genomes, the IL3000 Kenyan isolate [7,29], and the Tc1/148 Nigerian isolate [30,31]. Choosing the most adequate reference for the sample being analysed may potentially improve the VAPPER results by increasing mapping sensitivity. However, we have previously shown that closely related *T. congolense* strains (i.e. with short genetic distances) do not always have equally related *VSG* repertoires [18].

For *T. vivax*, the gVAP is based on presence or absence of pre-defined *VSG* genes, rather than the phylotype frequencies described for *T. congolense*. The *T. vivax VSG* repertoire is composed of distantly related lineages with sequence diversity as low as 40% [7]. These lineages are broadly conserved across isolates, which allow us to build a *VSG* database for the entire species. *VSG*-containing contigs are identified using BLAST 2.7.1 to detect sequence homology with a *T. vivax VSG* database. This information is added to a regularly updated presence/absence binary matrix of *T. vivax* genomes and applied to a Euclidean distance-based clustering analysis. The results are presented as a heatmap and dendrogram, putting the sample in the context of the available *T. vivax* genomes.

For *T. vivax* transcriptomic analyses (tVAP), VAPPER works similarly to *T. congolense*, but using a VSG database rather than protein motifs. As sequencing depth is generally not great enough to exhaustively detect all VSGs in a single sample, and to accommodate the substantial number of strain-specific VSGs, using the raw VSG database is not the most tractable approach. Therefore, to make the *T. vivax* transcriptomic analysis exhaustive and consistent with the *T. congolense* tVAP, we defined 174 phylotypes that combine VSGs with a sequence identity score of 70% or more. In the presence of low genome coverage, it is essential to adopt a phylotype-based system rather than dealing with individual genes.

In its Linux version, VAPPER can process multiple samples concurrently, providing that the input files are compiled in a single directory. Results are shown for all samples simultaneously, allowing direct comparison of variant antigen profiles across multiple isolates, conditions, or replicates. The tabular output can be incorporated in downstream statistical analysis, whilst the graphical outputs provide figures for the visualization of antigen repertoire variability.

Linux Package Installation

To facilitate usage, the installation of VAPPER and its dependencies is automated. Upon first download of the software, a single script will ensure the system has all the required dependencies and install them in a local directory if necessary. In naïve environments and for users without administrator rights to install the necessary libraries, a Python virtual environment can be set upon each new session. A step-by-step guide for the installation and usage of VAPPER can be found in Supplementary File 1.

The Galaxy Tool

VAPPER is available for installation in local Galaxy servers from the Galaxy ToolShed (https://toolshed.g2.bx.psu.edu/repository?repository_id=08b5616f1d3df20c). The purpose of the incorporation of VAPPER in Galaxy local servers is to provide a simple front-end component for non-experienced users (Fig. 2). Results can be visualised directly in Galaxy, or can be downloaded as a compressed folder containing an HTML file with combined results, individual PNG and PDF files of the heatmaps, PCA plots, and bar charts produced, and the CSV files containing the raw values of phylotype proportions and deviation from the mean. A step-by-step guide for the installation of VAPPER on a Galaxy local server can be found in Supplementary File 1.

Benchmarking

The performance of the *T. congolense* gVAP pipeline was compared to the manually annotated VAP of the IL3000 reference genome (Fig. 3A) and to the BLAST-based VAPs of 41 isolates (Fig. 3B) [18]. There is a very good correlation between profiles produced by VAPPER and the known IL3000 VAP (R^2 = 0.88, t(13) = 9.7321, P < 0.001) and a good correlation with the BLAST-based method (R^2=0.67, Pearson's product moment correlation, $t_{(566)=}$34.4, p < 0.001). Minor differences were further investigated and found to be due to BLAST's difficulty in either analysing small contigs or quantifying multiple VSGs in the same contig sequence. Therefore, in general, more VSGs were recovered with VAPPER than with BLAST (Mean ± σ=721±277 vs. 669 ± 292, paired *t*-test, *p*-value = 0.005). A further strength of VAPPER is the ability to deal with poor, fragmented, genome assemblies. As described in our previous paper [18], when a single *VSG* gene is located in two distinct contig fragments, BLAST counts them incorrectly as separate genes, whereas VAPPER will not because the diagnostic motif is only present once. Therefore, we can now accurately calculate antigen profiles from incomplete genome assemblies (up to 30%), and with a *VSG* fragmentation level up to 40% of the original gene length (223 nucleotides) (Fig. 3C).

Validation by example

T. congolense gVAP

We have used the VAPPER to analyse the genomic repertoire of 98 *T. congolense* samples of savannah and forest-subtypes, collected from 12 countries across Africa, and previously described by us [18] and others [26]. In Fig. 4, two heatmaps and corresponding dendrograms show how the VSG repertoires of each strain relate to each other. On the left, the heatmap represents phylotype proportion, i.e. how many genes a specific phylotype contains in the context of the complete *VSG* repertoire for

a given strain (Fig. 4A). This heatmap shows that P4, 8, 9, 10, and 14 have few genes in all strains, whereas other phylotypes (e.g. P1, 2, 15) are more variable, being quite abundant in some strains and rare in others. The heatmap on the right shows phylotype deviation from the mean (Fig. 4B), which is calculated as the difference between the phylotype proportion shown in panel A and the arithmetic mean of phylotype proportions. The latter is calculated from the current database, thus it will change as new samples are added.

The phylotype proportion variation patterns are perhaps better detected in the normalised heatmap (Fig. 4B). For example, it is possible to detect a signature of underrepresented P15 characteristic of all forest-subtype samples (denoted by “a”), abundant P15 in all Kenyan isolates (in purple), as well as a distinct pattern characteristic of strains IL3578 to IL2326, characterised by the combination of low P1 to 3 and high P7 (denoted by “b”). The latter does not seem to be related to geography, as it encompasses isolates from Kenya, Uganda, and Burkina Faso. The PCA plot further indicates that VSG repertoires and geography are only weakly correlated (Fig. 4C), which agrees with our previous observation that *T. congolense* VSG repertoires do not mimic either population structure or geography [18].

T. congolense tVAPs

We have used VAPPER to analyse the expressed VSG repertoire of the metacyclic (infective) life stage of *T. congolense.* For that, we have produced a tVAP for the strain TC13, whose transcriptome was published by Awuoche *et al.* (2018) [32]. We have compared the metacyclic tVAP of this strain with the 1/148 strain (MBOI/NG/60/1-148) that we have previously described [30]. Furthermore, we have compared them to the genomic *VSG* repertoires of the same strain, or a related one (Fig. 5). As we do not have a genome sequence for the TC13 isolate, we compared it to IL3000, which was isolated in the same region (Transmara, Kenya) [33].

When we compare the gVAPs of 1/148 and IL3000, we see that they are distinct, and so are the tVAPs (e.g. P4 is more represented in TC13, whereas P10 is more represented in 1/148 than in TC13). However, P8 is overrepresented in both isolates compared to the genomic repertoires (Fig. 5). This agrees with our previous observation that the pattern of metacyclic *VSG* expression is significantly different from the genome repertoires, and that the metacyclic *VSG* repertoire is particularly enriched for P8 genes [18]. With the analysis of the TC13 transcriptome, we can now add that this enrichment does not seem to be strain-specific, but rather equally applicable to *T. congolense* strains of distinct backgrounds.

T. vivax gVAP

The *T. vivax* gVAP shows the VAPs in the context of a *T. vivax* genome database. As proof of concept, we have produced VAPs for 11 isolates collected across Nigeria. The dendrogram represents the relationships between the multiple samples, whereas the heatmap shows whether *VSG* genes are present or absent in each of them (Fig. 6A). Overall, these profiles show high reproducibility across samples, as would be expected for isolates of similar geographical location. Yet, the profiles also reveal some differences amongst isolates, suggesting strain-specific variation and highlighting the potential epidemiological value of particular VSGs.

T. vivax tVAP

As proof of concept, we have used tVAP to obtain the expressed VAP of the previously published strain IL1392 [34] (Fig. 6B). We observe 21 phylotypes being expressed, of which 8 have a substantial weight. P2 is the most abundant phylotype, followed by P142 and P143. These profiles can be used to compare VSG repertoires and identify

phylotype patterns that may be epidemiologically relevant, perhaps contributing to the considerable phenotypic variation observed in *T. vivax* AAT.

We understand that the expansion of VAPPER to the widely studied, human-infective species, *T. brucei* will have great value to the community. Current *T. brucei* VSG analyses are extensive and thorough, but strain-specific because the extremely dynamic, highly recombinant VSG repertoire is a challenge for profiling approaches. This task will be possible in the future, but will definitely require a novel methodology. Attempts to profile VSGs based on amino acid signatures such as the one presented here for *T. congolense* will likely fail due to the extreme degree of mosaicism [35] and the ability to convert genes between very diverse donor regions [36]. If a minimal VSG recombination unit can be determined, alternative systematics may resort to mosaic frequencies, particularly because mosaics formed from the same set of genes can have higher nucleotide identity between themselves than their precursors. In fact, alternative antigenic profiling methods already exist for some organisms. For example, profiling of *Plasmodium falciparum var* gene diversity was achieved through a population genomic framework [37] targeting variation in the Duffy binding-like alpha (DBLα) motif, an ubiquitous 500 nucleotide fragment marker. Yet, for other pathogens with variant multi-copy gene families, such as *Trypanosoma cruzi*, antigen profiling has not yet been done. For these, species- and gene family-specific motif-based approaches, such as the one presented here for *T. congolense* may offer a tractable solution.

Conclusion

VAPPER is the first tool for the systematic analysis of *VSG* gene and expression diversity across strains and during infections. It establishes a practical approach for measuring antigenic diversity in these important pathogens based on universal protein

motifs and/or gene mapping. VAPPER allows us to identify and characterise differences in antigenic repertoires between strains, hosts, and conditions, which may be the starting point to build a real understanding of the association between parasite genotypes and outcomes of AAT.

Availability and requirements

Project name: VAPPER – High-throughput Variant Antigen Profiling in African trypanosomes

Project home page: https://github.com/PGB-LIV/VAPPER

Operating System: Platform independent

Programming language: Python

Installation Requirements: Velvet 1.2.10; HMMER 3.1b2; Bowtie 2 2.2.6; SAMtools 1.6; Cufflinks 2.2.1; BLAST 2.7.1; EMBOSS

License: Apache v.2.0

RRID: SCR_016993

Availability of supporting data

Snapshots of our code and other data further supporting this work are available in the GigaScience repository, GigaDB [39].

Figure Legends

Figure 1 Methodological workflow according to species (*T. congolense* or *T. vivax*) and input data [genomic (gVAP) or transcriptomic (tVAP)].

Figure 2 Screenshot of VAPPER on the Galaxy interface. This interface is available after installation of VAPPER from the Galaxy ToolShed [20] into a local Galaxy server. In this case, VAPPER was installed on the University of Liverpool Galaxy server. The blue panel on the right shows how to search and select VAPPER after installation. The

white panel at the centre shows the options available for the user, including the prefix name of the sample to appear on the output figures, the species, and the type of input data. If any genomic pipeline is selected, further options for genome assembly parameters are available. Finally, the user can choose whether to get the graphs in PDF format (default is PNG only).

Figure 3 VAPPER performance (*T. congolense* genomic pipeline). (A) Correlation of phylotype frequencies produced by VAPPER and those manually curated in the *T. congolense* IL3000 reference genome sequence [7]. Pearson's product moment correlation statistics: $R^2 = 0.88$, $t(13) = 9.7321$, $P < 0.001$. (B) Correlation of phylotype frequencies produced by VAPPER and BLAST-based [38] phylotype frequencies in a panel of 41 *T. congolense* strains. Pearson's product moment correlation: $R^2 = 0.64$, $t(566) = 34.39$, $P < 0.001$. Phylotypes are color-coded according to the key. (C) VAPPER accuracy in fragmented (red) or incomplete (blue) genomes. Line graphs show correlations of the expected antigen profiles of a known set of VSGs sequences from the IL3000 genome sequence with antigen profiles produced from fragmented VSGs or incomplete VSG repertoires. Fragmentation and genome incompletion were simulated from random sampling. Gene fragmentation was calculated as a proportion of the mean length of the original VSG sequences (Mean±σ=1163±129 nucleotides). Figure adapted from [18].

Figure 4 VAPPER output for *T. congolense* genomic pipeline. (A) Heatmap and corresponding dendrogram showing the variant antigen profiles (VAP) of the current genomic database expressed as phylotype frequencies [18,26]. (B) Heatmap and corresponding dendrogram showing the variant antigen profiles (VAP) of the current genomic database expressed as deviation from the mean phylotype frequency [18,26]. Labels "a" and "b" are referred to in the text. (C) PCA plot representing variation in *VSG* repertoire across the *T. congolense* genomic database [18,26] (N=97).

Figure 5 VAPPER output for *T. congolense* transcriptomic pipeline. Stacked bar charts showing expressed variant antigen profiles (VAPs) of metacyclic-stage *T. congolense* from strain 1/148 [18] and TC13 [32] compared to the genomic repertoires of the same strain (1/148) or a closely related one (IL3000) [29]. Phylotypes are colour-coded according to key. Size of each stack represents proportion of the phylotype relative to the total repertoire of expressed *VSGs*.

Figure 6 VAPPER output for *T. vivax* genomic and transcriptomic pipelines. (A) Heatmap and corresponding dendrogram showing *T. vivax* variant antigen profiles (VAPs) of 11 Nigerian strains. (B) Expressed VAP of *T. vivax* IL1392 bloodstream form previously published by Jackson *et al.* (2015) [34], shown as a bar chart.

Declarations

Ethics approval and consent to participate

Not applicable.

Consent for publication

Not applicable.

Competing interests

The authors declare that they have no competing interests.

Funding

This work was supported by a Grand Challenges (Round 11) award from the Bill and Melinda Gates Foundation, a BBSRC New investigator Award (BB/M022811/1), and the Technology Directorate of the University of Liverpool to APJ.

Authors' contributions

SSP wrote the original code in Perl and tested the software. JH and ARJ wrote the final code in Python. SSP and APJ conceptualized the software and wrote the manuscript. All authors contributed to and approved the final manuscript.

References

1. Barry JD, Ginger ML, Burton P, McCulloch R. Why are parasite contingency genes often associated with telomeres? Int. J. Parasitol. 2003;33:29–45.

2. de la Fuente J, Lew A, Lutz H, Meli ML, Hofmann-Lehmann R, Shkap V, et al. Genetic diversity of anaplasma species major surface proteins and implications for anaplasmosis serodiagnosis and vaccine development. Anim. Health Res. Rev. 2005;6:75–89.

3. Kyes SA, Kraemer SM, Smith JD. Antigenic variation in Plasmodium falciparum: Gene organization and regulation of the var multigene family. Eukaryot. Cell. 2007;6:1511–20.

4. McCulloch R, Cobbold CA, Figueiredo L, Jackson A, Morrison LJ, Mugnier MR, et al. Emerging challenges in understanding trypanosome antigenic variation. Emerg. Top. Life Sci. 2017;1:585–92.

5. Pays E. The variant surface glycoprotein as a tool for adaptation in African trypanosomes. Microbes Infect. 2006;8:930–7.

6. Cross G a M, Kim HS, Wickstead B. Capturing the variant surface glycoprotein repertoire (the VSGnome) of Trypanosoma brucei Lister 427. Mol. Biochem. Parasitol. Elsevier B.V.; 2014;195:59–73.

7. Jackson AP, Berry A, Aslett M, Allison HC, Burton P, Vavrova-Anderson J, et al. Antigenic diversity is generated by distinct evolutionary mechanisms in African trypanosome species. Proc. Natl. Acad. Sci. U. S. A. 2012;109:3416–21.

8. Matthews KR, McCulloch R, Morrison LJ. The within-host dynamics of African

trypanosome infections. Philos. Trans. R. Soc. Lond. B. Biol. Sci. 2015;370:20140288-.

9. Capewell P, Clucas C, DeJesus E, Kieft R, Hajduk S, Veitch N, et al. The TgsGP gene is essential for resistance to human serum in Trypanosoma brucei gambiense. PLoS Pathog. 2013;9:e1003686.

10. Uzureau P, Uzureau S, Lecordier L, Fontaine F, Tebabi P, Homblé F, et al. Mechanism of Trypanosoma brucei gambiense resistance to human serum. Nature. 2013;501:430–4.

11. De Greef C, Hamers R. The serum resistance-associated (SRA) gene of Trypanosoma brucei rhodesiense encodes a variant surface glycoprotein-like protein. Mol. Biochem. Parasitol. 1994;68:277–84.

12. Van Xong H, Vanhamme L, Chamekh M, Chimfwembe CE, Van Den Abbeele J, Pays A, et al. A VSG expression site-associated gene confers resistance to human serum in Trypanosoma rhodesiense. Cell. 1998;95:839–46.

13. Wiedemar N, Graf FE, Zwyer M, Ndomba E, Kunz Renggli C, Cal M, et al. Beyond immune escape: a variant surface glycoprotein causes suramin resistance in Trypanosoma brucei. Mol. Microbiol. 2018;107:57–67.

14. Salmon D, Geuskens M, Hanocq F, Hanocq-Quertier J, Nolan D, Ruben L, et al. A novel heterodimeric transferrin receptor encoded by a pair of VSG expression site-associated genes in T. brucei. Cell. 1994;78:75–86.

15. Marcello L, Menon S, Ward P, Wilkes JM, Jones NG, Carrington M, et al. VSGdb: A database for trypanosome variant surface glycoproteins, a large and diverse family of coiled coil proteins. BMC Bioinformatics. 2007;8:1–8.

16. Weirather JL, Wilson ME, Donelson JE. Mapping of VSG similarities in Trypanosoma brucei. Mol. Biochem. Parasitol. 2012;181:141–52.

17. Mugnier MR, Cross GAM, Papavasiliou FN. The in vivo dynamics of antigenic variation in Trypanosoma brucei. Science (80-.). 2015;347:1470–3.

18. Silva Pereira S, Casas-Sanchez A, Haines LR, Absolomon K, Ogugo M, Sanders

M, et al. Variant antigen repertoires in Trypanosoma congolense populations and experimental infections can be profiled from deep sequence data with a set of universal protein motifs. Genome Res. 2018;28:1383–94.

19. Wang CW, Lavstsen T, Bengtsson DC, Magistrado PA, Berger SS, Marquard AM, et al. Genetic diversity of expressed Plasmodium falciparum var genes from Tanzanian children with severe malaria. Malar. J. Malaria Journal; 2012;11:230.

20. Blankenberg D, Von Kuster G, Bouvier E, Baker D, Afgan E, Stoler N, et al. Dissemination of scientific software with Galaxy ToolShed. Genome Biol. 2014.

21. Afgan E, Baker D, van den Beek M, Blankenberg D, Bouvier D, Čech M, et al. The Galaxy platform for accessible, reproducible and collaborative biomedical analyses: 2016 update. Nucleic Acids Res. 2016;44:W3–10.

22. Cock PJA, Fields CJ, Goto N, Heuer ML, Rice PM. The Sanger FASTQ file format for sequences with quality scores, and the Solexa/Illumina FASTQ variants. Nucleic Acids Res. 2009;38:1767–71.

23. Pearson WR, Lipman DJ. Improved tools for biological sequence comparison. Proc. Natl. Acad. Sci. 1988;85:2444–8.

24. Zerbino DR. Using the Velvet de novo assembler for short-read sequencing technologies. Curr. Protoc. Bioinforma. 2010.

25. Eddy SR. A new generation of homology search tools based on probabilistic inference. Genome Inform. 2009;23:205–11.

26. Tihon E, Imamura H, Dujardin J-C, Van Den Abbeele J, Van den Broeck F. Discovery and genomic analyses of hybridization between divergent lineages of *Trypanosoma congolense* , causative agent of Animal African Trypanosomiasis. Mol. Ecol. 2017;

27. Langmead B, Salzberg SL. Fast gapped-read alignment with Bowtie 2. Nat Methods. 2012;9:357–9.

28. Trapnell C, Roberts A, Goff L, Pertea G, Kim D, Kelley DR, et al. Differential gene and transcript expression analysis of RNA-seq experiments with TopHat and

Cufflinks. Nat. Protoc. 2012;7:562–78.

29. Gibson W. The origins of the trypanosome genome strains Trypanosoma brucei brucei TREU 927, T. b. gambiense DAL 972, T. vivax Y486 and T. congolense IL3000. Parasit. Vectors. BioMed Central Ltd; 2012;5:71.

30. Young CJ, Godfrey DG. Enzyme polymorphism and the distribution of Trypanosoma congolense isolates. Ann. Trop. Med. Parasitol. 1983;77:467–81.

31. Abbas AH, Pereira SS, D'Archivio S, Wickstead B, Morrison LJ, Hall N, et al. The structure of a conserved telomeric region associated with variant antigen loci in the blood parasite <i>Trypanosoma congolense<i/>. Genome Biol. Evol. 2018;evy186.

32. Awuoche EO, Weiss BL, Mireji PO, Vigneron A, Nyambega B, Murilla G, et al. Expression profiling of Trypanosoma congolense genes during development in the tsetse fly vector Glossina morsitans morsitans. Parasit. Vectors. Parasites & Vectors; 2018;11:1–18.

33. Ferrante A, Allison AC. Alternative pathway activation of complement by African trypanosomes lacking a glycoprotein coat. Parasite Immunol. 1983;5:491–8.

34. Jackson AP, Goyard S, Xia D, Foth BJ, Sanders M, Wastling JM, et al. Global Gene Expression Profiling through the Complete Life Cycle of Trypanosoma vivax. PLoS Negl. Trop. Dis. 2015;9:e0003975.

35. Marcello L, Barry JD. Analysis of the VSG gene silent archive in Trypanosoma brucei reveals that mosaic gene expression is prominent in antigenic variation and is favored by archive substructure. Genome Res. 2007;17:1344–52.

36. Hall JPJ, Wang H, Barry JD. Mosaic VSGs and the Scale of Trypanosoma brucei Antigenic Variation. Horn D, editor. PLoS Pathog. 2013;9:e1003502.

37. Barry AE, Leliwa-Sytek A, Tavul L, Imrie H, Migot-Nabias F, Brown SM, et al. Population genomics of the immune evasion (var) genes of Plasmodium falciparum. PLoS Pathog. 2007;3:1–9.

38. Altschul SF, Gish W, Miller W, Myers EW, Lipman DJ. Basic local alignment search tool. J. Mol. Biol. 1990;215:403–10.

39. Silva Pereira S; Heap J; Jones AR; Jackson AP: Supporting data for "VAPPER: High-throughput Variant Antigen Profiling in African trypanosomes" GigaScience Database. 2019. http://dx.doi.org/10.5524/100626.

Figure 1

Click here to access/download;Figure;Figure1.pdf

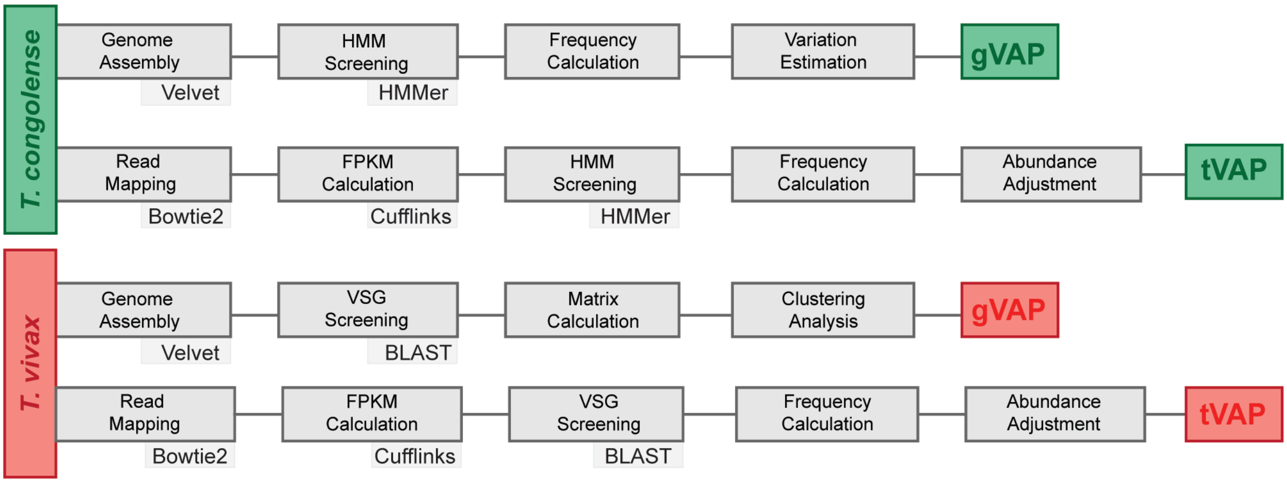

Figure 2

Click here to access/download;Figure;Figure2.pdf

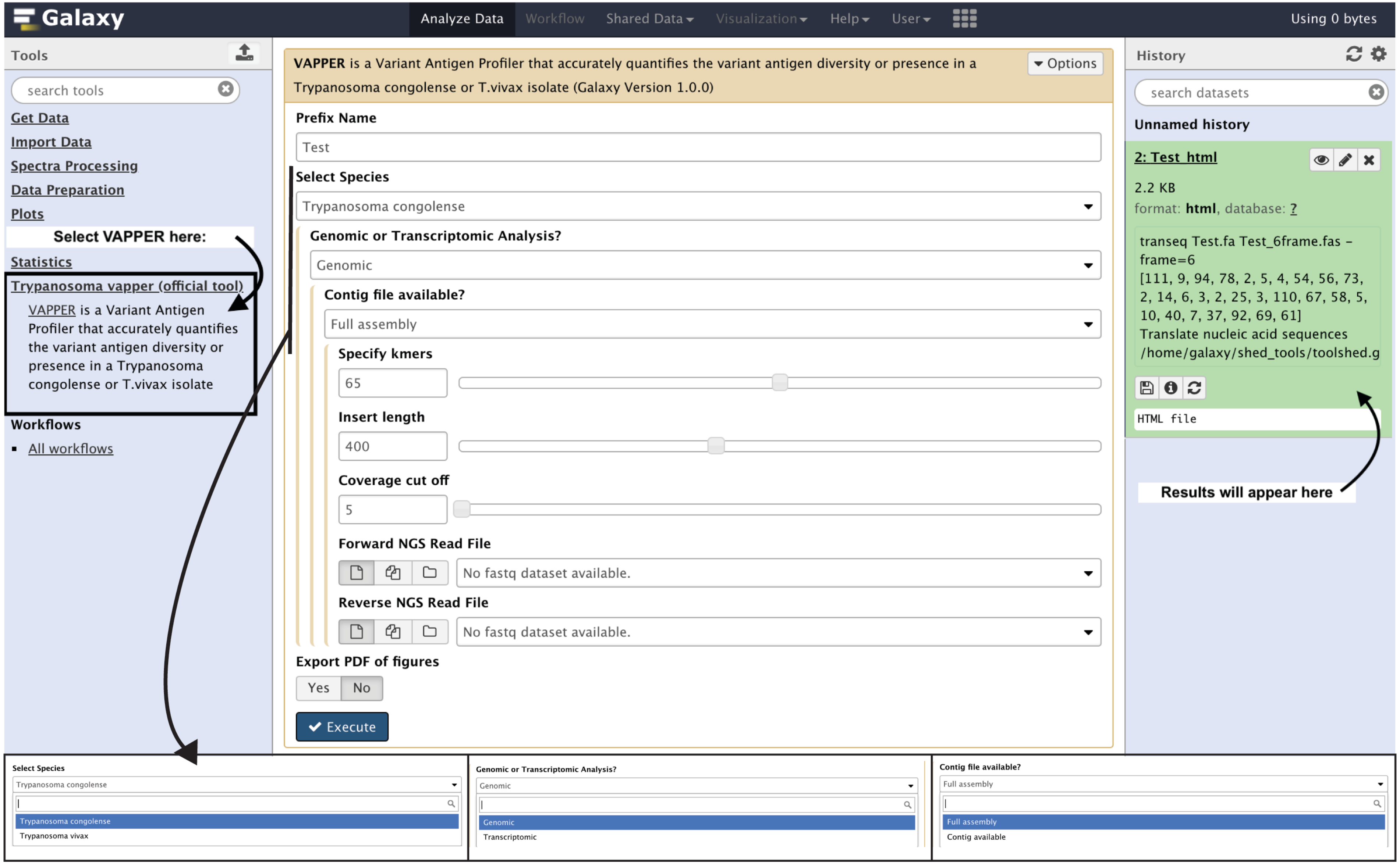

Figure 3 Click here to access/download;Figure;Figure3.pdf

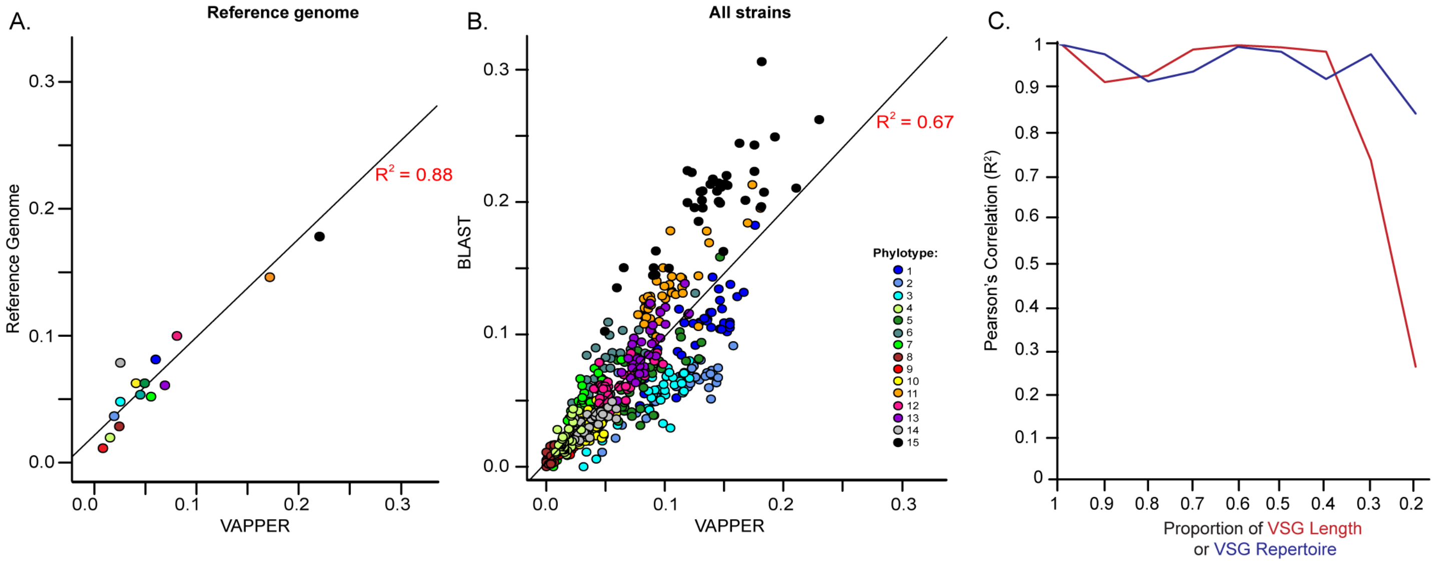

Figure 4

Click here to access/download;Figure;Figure4.pdf

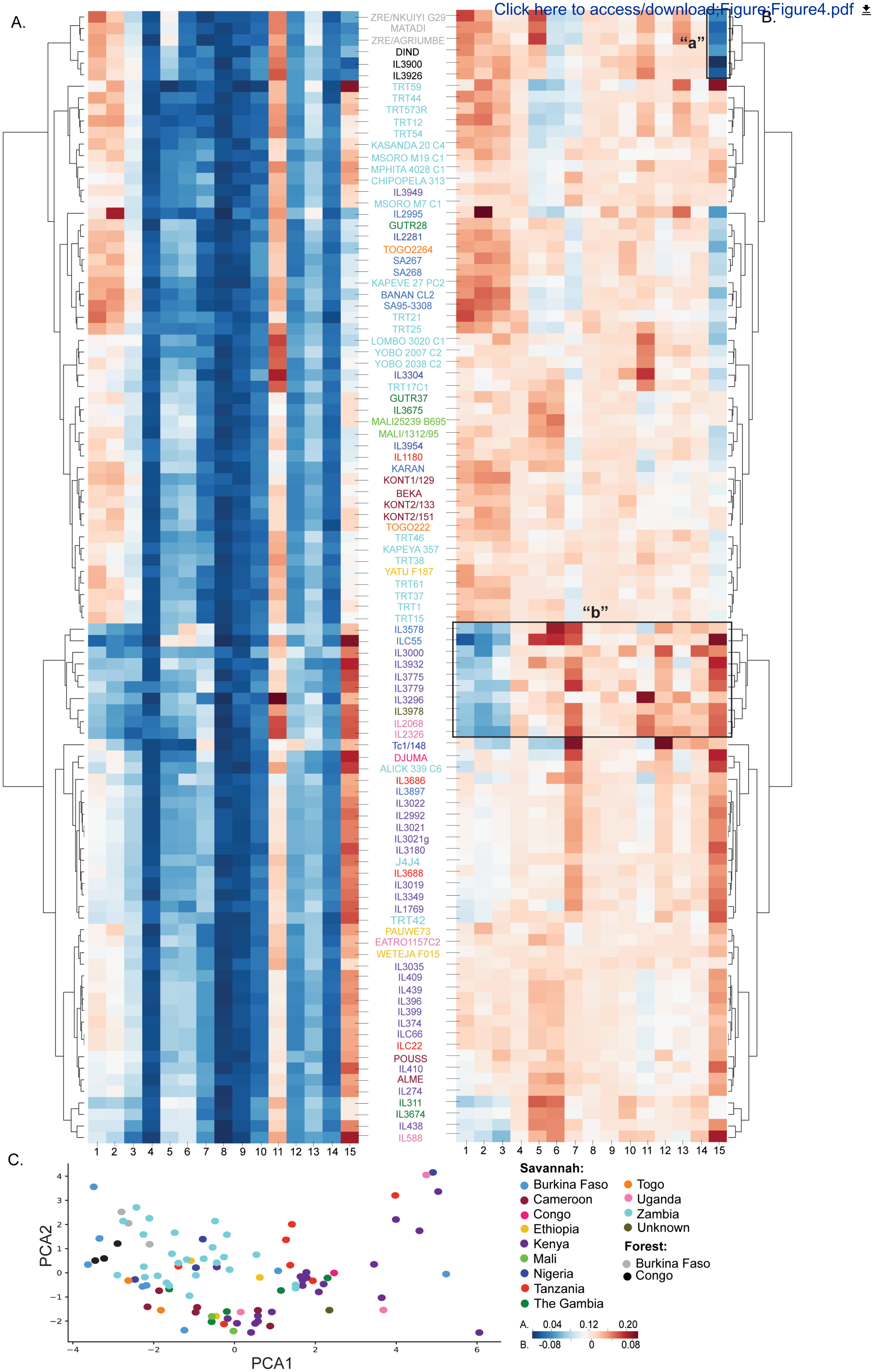

Click here to access/download;Figure;Figure5.pdf

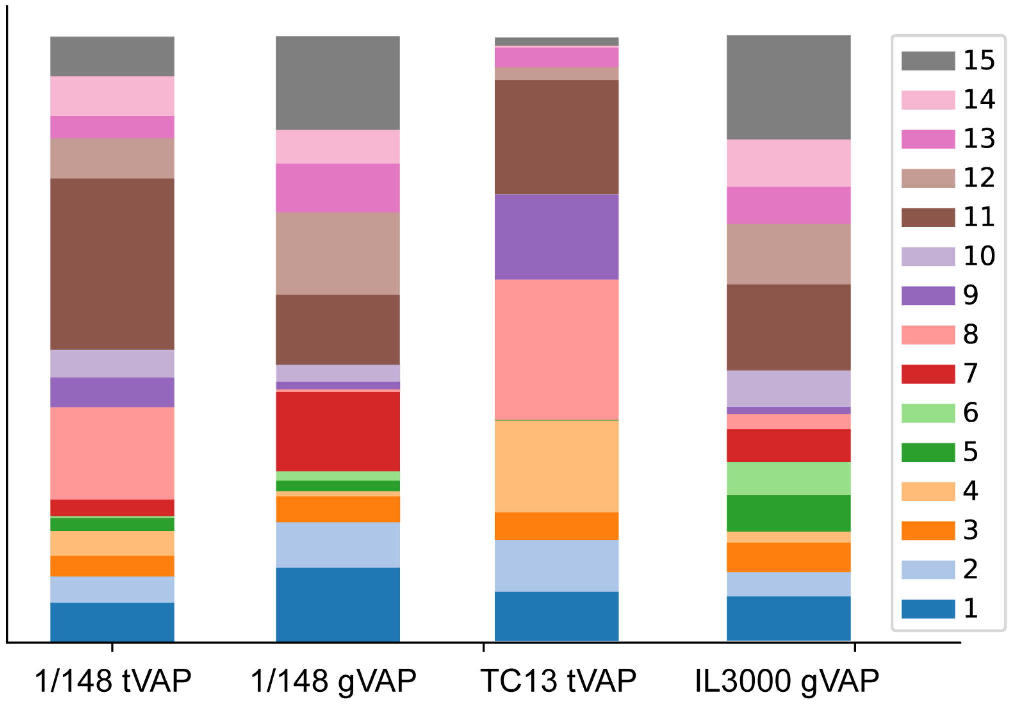

Figure 6

Click here to access/download;Figure;Figure6.pdf

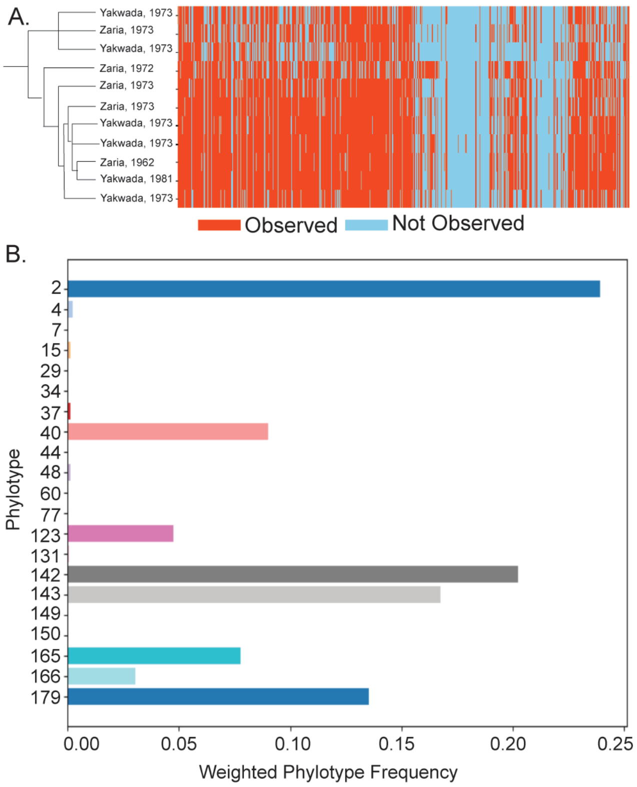

Supplementary File 1

Click here to access/download
Supplementary Material
SupplementaryFile1_VAPPER_User_Guide.docx
