# Supplementary material for: VAPPER: High-throughput variant antigen profiling in African trypanosomes of livestock
Source: Gigascience. 2019 Aug 29;8(9):giz091. doi: 10.1093/gigascience/giz091 (PMC6735694; doi:10.1093/gigascience/giz091)
Supplement: giz091_Supplemental_File [file giz091_supplemental_file.docx]

**VAPPER User Guide**

# Standalone version

**Requirements:** Python 2.7 + seaborn version 0.8.0

Vap.py is a master python script that- parses command line parameters and selects pathways accordingly. It imports files Tryp_G.py for the T. congolense genomic pipeline, Tryp_T.py for the T. congolense transcriptomic pathway, and Tryp_V.py for the T. vivax analysis. It can will import Tryp_Multi.py if managing multiple samples at once.

**Packages used by VAPPER**

| Package | Version | Website |
| --- | --- | --- |
| Velvet | 1.2.10 | <https://www.ebi.ac.uk/~zerbino/velvet/> |
| EMBOSS transeq | 6.6.0.0 | <http://emboss.open-bio.org/> |
| Bowtie 2 | 2.2.6 | <http://bowtie-bio.sourceforge.net/bowtie2/index.shtml> |
| HMMER | 3.1.b2 | <http://hmmer.org/> |
| blast | 2.7.1 | <https://blast.ncbi.nlm.nih.gov/Blast.cgi?CMD=Web&PAGE_TYPE=BlastDocs&DOC_TYPE=Download> |

**Installation**

To ensure your system has all the required dependencies and before running the VAPPER code for the first time, please type:

source install.sh

This script will:

1. Temporarily add a path to your system PATH variable

2. Check for the installation of transeq a required EMBOSS application

3. If absent, it will download and install it

4. Then, in the virtual environment, VAPENV, it will install any required python packages

This only needs to be done **once per installation**. However upon each new session before calling VAPPER it will be necessary to **set the $PATH and the virtual environment again** by typing:

source setup.sh

**Usage**

Vap.py [-h] [-s S] [-con CON] [-t] [-p] [-strain STRAIN] [-dir DIR] [-cdir CDIR]

[-f F] [-r R] [-k K] [-i I] [-cov COV] name

python Vap.py –help

- Lists the command line arguments accepted as below.

Positional and Optional Arguments:

| **Name** | **Prefix for results directory and files therein** |
| --- | --- |
| -s, -S | Species: T.congolense (default) or T.vivax |
| -con | Contigs file (fasta) |
| -t, -T | T. congolense Transcriptomic Pathway |
| -p, -P | Export PDFs of images to results directory (default is PNGs only) |
| -strain | Strain for Transcriptomic pathway (defaults to Tc148) |
| -dir | Directory that holds multiple paired NGS readfiles for analysis |
| -cdir | Directory that holds multiple pre-assembled contigs (fasta) files for analysis |
| -f | Forward NGS Read File |
| -r | Reverse NGS Read File |
| -k | kmers for de novo assembly (default = 65) |
| -i | Insert Length for de novo assembly (default = 400) |
| -cov | Coverage cut off for de novo assembly (default = 5) |

**Examples of Use**

1. ***T. congolense* Genomic pathway:**
2. Single sample of *T. congolense* from paired NGS read files.

$ python Vap.py sgtest -f Test1.fastq -r Test2.fastq

Result images, csv files and html file will be found in directory results/sgtest/

1. Multiple sample of *T. congolense* from several sets of paired NGS read files placed in directory /mydata/

Each set of paired files should have the same name except for trailing 1 or 2 (e.g. Test1.fastq, Test2.fastq)

$ python Vap.py mgtest -dir mydata

Result images, csv files and html file will be found in directory results/mgtest/

1. Single sample of T. congolense from a contigs file

$ python Vap.py sctest -con Test.fa

Result images, csv files and html file will be found in directory results/sctest/

1. Multiple sample of *T.congolense* from several contigs file (*.fa) placed in directory mycdata

$ python Vap.py mctest -cdir mycdata

Result images, csv files and html file will be found in directory results/mctest/

1. ***T. congolense* Transcriptomic pathway**
2. Single sample of *T. congolense*, transcriptomic pathway from paired Transcript read files

$ python Vap.py sttest -t -f Transcripts.1 -r Transcripts.2

Result images, csv files and html file will be found in directory results/sttest/

1. Multiple sample of *T. congolense* from several sets of paired transcript read files place in directory /mytdata/

Each set of paired files should have the same name except for trailing 1 or 2 (eg Transcripts.1, Transcripts.2)

$ python Vap.py mttest -t -dir mytdata

Result images, csv files and html file will be found in directory results/mttest/

1. **T. vivax Genomic pathway:**
2. Single sample of *T. vivax* from paired NGS read files.

$ python Vap.py svtest -s T.vivax -f Test1.fastq -r Test2.fastq

Result images, csv files and html file will be found in directory results/svtest/

1. Multiple sample of *T. vivax* from several sets of paired NGS read files place in directory /myvdata/

Each set of paired files should have the same name except for trailing 1 or 2 (eg Test1.fastq, Test2.fastq)

$ python Vap.py mvtest -s T.vivax -dir myvdata

Result images, csv files and html file will be found in directory results/mvtest/

1. Single sample of T.vivax from a contigs file

$ python Vap.py scvtest -s T.vivax -con Test.fa

Result images, csv files and html file will be found in directory results/scvtest/

1. Multiple sample of *T. vivax* from several contigs file (*.fa) placed in directory mycdata

$ python Vap.py mcvtest -s T.vivax -cdir mycdata

Result images, csv files and html file will be found in directory results/mcvtest/

1. ***T. vivax* Transcriptomic pathway:**
2. Single sample of *T. vivax* from paired NGS read files.

$ python Vap.py stvtest -s T.vivax -t -ref reference.fasta -f transcripts.1 - r transcripts.2

Results image, html and .csv file will be found in results/stvtest/

1. Multiple samples of *T. vivax* from several sets of pair NGS reads placed in directory /myvdata/

$ python Vap.py mtvtest -s T.vivax -t -ref reference.fasta -dir myvdata

Results image, html and .csv files will be found in results/mtvtest/

The user-supplied reference file (reference.fasta) should contain assembled transcripts. We advise it to be created using Trinity (Grabherr et al. 2013).

**Test Data:**

The directory "Example_data" contain examples of the outputs that should be expected. For *T. congolense*, this includes two PDF and PNG heatmaps/dendrograms; a PCA plot; and two CSV files containing the VAP of a test sample, expressed as the phylotype relative frequency and variation (the deviation from the mean). For *T. vivax*, this includes a cluster map in the form of heatmap/dendrogram, and a CSV file with a binary matrix representing a VAP of a test sample. Additionally, we have provided a small test contig file (to keep file size manageable):

$ python Vap.py tc_test -con test_data/Tc_contigs.fa

Results will be found in results/tc_test.

# Galaxy version

Below are the steps for Galaxy installation on a local server and VAPPER installation from the Toolshed. This has been tested on a new Ubuntu 64-bit Linux installation. For more detail, visit <https://galaxyproject.org/admin/get-galaxy/>.

**Install Galaxy**

1. Get latest version of Galaxy

$ git clone -b release_18.09 <https://github.com/galaxyproject/galaxy.git>

1. Start off Galaxy

$ cd galaxy

$ sh run.sh

This will take some time when run for the first time. Once complete, the last output to Terminal should be:

$ Starting server in PID 5759

$ serving on <http://localhost:8080>

Galaxy should now be available via your browser at <http://localhost:8080>. Go there and register yourself as a user.

1. Obtain administrator rights

You now need to make yourself an administrator, this involves editing the galaxy config file.

1. Stop galaxy by returning to the terminal and pressing ^C [Ctrl and C together]
2. In the terminal go to the galaxy directory

$ cd galaxy

1. Copy the sample config file to an actual config file

$ cp config/galaxy.yml.sample config/galaxy.yml

1. Edit the config file (I use 'nano')

$ nano galaxy.yml

1. Find admin_users

$ ^w admin_users

1. De-comment the line #admin_users: '' and add your galaxy log in email address. For example:

admin_users: [user1@example.com](mailto:user1@example.com)

1. Save the file and exit
2. Restart the galaxy server again

$ sh run.sh

(it should be quicker this time)

Browse to <http://localhost:8080> and log in. The 'Admin' option should now be available in the top Navigation bar: Click it

**Install VAPPER**

1. Select the option “Install new tools” from the side bar
2. Click on the only repository listed: ‘Galaxy Main Tool Shed’
3. Search for VAPPER
4. Press enter

On the Valid Repositories page:

1. Select Vapper Preview and Install
2. Click the tab at the top ‘Install to Galaxy’

This will produce a warning page listing all the tool dependencies that are not available (8 if using a fresh Linux environment).

1. Click install for the server to install these dependencies
2. In the 'Add new tool panel section:', type: Trypanosoma
3. Click Install

It should then go to a 'Monitor installing tool shed repositories' page, where status will show as “Cloning”. This will then update to Installing tool dependencies (this takes a while). Once it is complete:

1. Press refresh to see 'Installed tool shed repositories' page
2. Return to the home page (for example, by clicking on the Galaxy logo on the top nav-bar). The new tool section should be visible

Before running the tool you need to get some data into your history. For example, Test.fa (T.congolense Fasta contig file), also available in ‘test-data’ directory on the VAPPER tool.

1. To get it into your history, click on ‘Get Data’ in the left nav column
2. Click on upload file
3. Drag and drop or ‘Choose local file’ and click start to upload the file to the server. (very quick as actually only copying it)

It should then be in your history

1. Select the Vapper job from the Trypanosoma section
2. Set up as follows:

Species: Trypanosoma congolense

Genomic or Transcriptomic: Genomic

Contig file available?

Contig available

Contig file: Test.fa

1. Click execute.

This job only takes a couple of minutes to run. If it is successful, VAPPER is installed correctly.

# References:

Silva Pereira, S. *et al.* (2018) Variant antigen repertoires in *Trypanosoma congolense* populations and experimental infections can be profiled from deep sequence data with a set of universal protein motifs. Genome Research 28: 1383-1394.

Tihon, E. *et al.* (2017) Discovery and genomic analyses of hybridization between divergent lineages of *Trypanosoma congolense*, causative agent of Animal African Trypanosomiasis. Mol Ecol. 26(23):6524-6538.
