# Supplementary material for: VAPPER: High-throughput variant antigen profiling in African trypanosomes of livestock
Source: Gigascience. 2019 Aug 29;8(9):giz091. doi: 10.1093/gigascience/giz091 (PMC6735694; doi:10.1093/gigascience/giz091)
Supplement: giz091_Response_to_Reviewer_Comments_Original_Submission [file giz091_response_to_reviewer_comments_original_submission.pdf]

Author's Response To Reviewer Comments

Close

17th June 2019

Dear Editor,

Re:. GIGA-D-18-00480 - VAPPER: High-throughput Variant Antigen Profiling in African trypanosomes of Livestock

We are grateful to you for the chance to resubmit our manuscript to GigaScience. We note that both reviewers are content that our main observations and interpretation. The comments from each reviewer were most valuable. Particularly, by addressing the concerns of Reviewer 1, we have improved the manuscript and increased the usability of the tool by providing an additional pipeline for the analysis of Trypanosoma vivax transcriptomic data (with an example shown in Figure 6B) and more description of installation and usage procedures. The latter has been addressed by carefully preparing a step-by-step user guide. Please note that we have changed Figure 6A, which previously showed a collection of isolates from across Africa, to show a more densely sampled set of same Nigerian location over multiple time points. We think this highlights the reproducibility of VAPPER in the best possible way, and shows how VAPPER can be used to provide epidemiological data within an authentic disease setting. Furthermore, and as suggested, we have included discussion of the scope of the tool and the importance of variant antigen profiles. Our responses to each specific comment are given below.

Responses to Reviewers' Comments:

Reviewer #1:
This is a useful tool for analysis of VSGs in trypanosomes of veterinary importance. The tool will support the analysis of VSG gene families which is challenging due to their abundance, mutation and/or recombination rates. A concern is VAPPER limitation to trypanosomes of veterinary importance, and thus the preliminary character of this work. It would be relevant to have it available to other trypanosomes, e.g. T. brucei brucei. Also, T. brucei that infect humans (e.g. T. brucei gambiense or rhodesiense), especially given the number of transcriptomes and genomes available for these parasites and the ability to track changes in VSG repertoire during infections with sequencing technologies. Also, it seems preliminary that the authors did not include validation of their tool with transcriptome of T. vivax, which is due to the lack of enough (as indicated by authors) transcriptome data. This work deserves to be published; however, it would be advantageous to include the option for analysis of variant antigens of other pathogens, at least the closest related T. brucei and additional validation to be complete.

Specific comments:
The limitation of the tool for two pathogens is discouraging, and I am astonished that the software did not include analysis of VSGs from T. brucei spp. which causes animal and human African trypanosomiasis. The authors also did not justify why they are not including an analysis of T. brucei spp. VSG genes since there is potential scientific interest from those working in the T. brucei field. Also, the implementation of VAPPER for analysis of other large antigen gene families such as those in T. cruzi, Leishmania, and Plasmodium etc was not included or discussed.

R.
We would like to thank reviewer 1 for his comments. We have carefully considered them and will address them individually below.

My suggestions are:
1) Authors should address why VAPPER does not analyze multigene families of other pathogens, and it should add the option to analyze (at least) T. brucei VSGs. The title of the manuscript and its introduction is suggestive of a broad utility of the tool for African trypanosomes, and not T. vivax and T. congolense only. This only became apparent much later in the text.

R.
Please be assured that including a T. brucei Variant Antigen Profile within VAPPER is a priority for us. Its omission is certainly not an oversight. The problem is that antigenic diversity in T. brucei is much more complex than T. congolense and T. vivax, and the computational solution to comparing any set of T. brucei strains is not yet available. Nevertheless, since T. brucei typically contributes less than 5% of cattle infections and there are many experiments now being devoted to T. congolense and T. vivax, we feel that there is worth in releasing the existing tools.

VSGs are refractory to conventional techniques of gene mapping because they recombine more than a normal gene. In T. congolense, this recombination is restricted to genes of the same cohort (or phylotype); therefore, we could design phylotype-specific protein motifs that are universal to any T. congolense strain. In T. vivax, the VSG recombination rate is generally low, which allows us to find the same gene across species; therefore, we could design a strategy based on clusters of gene orthologs. This is not the case in T. brucei; the VSG repertoire in this species is extremely dynamic, and virtually every VSG gene is a mosaic, likely to be found in other strains as a number of dispersed fragments around the genome. Very few T. brucei VSG are routinely found as orthologs across diverse strains.

So, while the reviewer correctly mentions that there are plenty of T. brucei genomes and transcriptomes available and that we can track changes in VSG repertoire during infections with current sequencing technologies, there are considerable problems to comparing VSG repertoire across multiple T. brucei strains. This is the main goal of VAPPER, producing a VAP for any isolate(s). Every T. brucei VSG analysis done to date has been developed on single strains, usually the same lab strain or its descendants, and VSG nomenclature for most parts has been study-specific. The picture is very different when we compare different clinical strains; then there is very little orthology and certainly mapping VSG reads from a clinical isolate onto a reference genome would make little sense. This dynamism and low sequence orthology between isolates mean that developing a VSG systematics universal to all T. brucei strains is an enormous challenge. We have included a paragraph discussing this and proposing alternative methodologies that may work best for this pathogen (ll. 279-289). We also address established variant antigen profiling methods in other pathogens (e.g. P. falciparum) and how a similar motif-based, but gene family-specific methodology could be developed for T. cruzi (ll. 289-297).

For clarity regarding the scope of VAPPER, we have changed the title, the abstract, and introduction to reiterate that VAPPER is a tool for the most prevalent livestock trypanosomes, T. congolense and T. vivax.

2) Validation of tVAP for T. vivax was not done due to the lack of transcriptomic data. How much data is needed for validation of this part of the tool? Because tVAP analysis for T. vivax uses a different pipeline than that of T. congolense, the validation of T. congolense does not guarantee that VAPPER would perform well for T. vivax tVAP. Can the authors generate the data to test the tool? This is another limitation of the tool which indicates that the work is still preliminary, and the authors should consider some additional effort to conclude this work prior publication. I do encourage the authors to do so, as VAPPER has the potential to be of interest in the parasitology community.

R.
We have developed (ll. 159-167) and validated (ll. 271-277) tVAP for T. vivax and included it in the revised VAPPER package.

Minor comments:
1) The author should include in the introduction why is relevant to study the variant antigen profile of a pathogen and indicate examples. That VSGs are essential for immune evasion is clear, but it is not so clear the advantages (or when it would be necessary) to study the whole repertoire of VSGs and separate them in distinct clades (or groups). One possibility is that the analysis of variant antigen repertoires over an infection, which might provide insights into recombination mechanisms or other processes. Although some examples are included in the Results, it would be good to have examples of potential applications in the Introduction.

R.

We have introduced this in the introduction (ll. 98-105).

2) I found it difficult to install the tool using galaxy, and I did not find much instruction on how to proceed with this in the manuscript. I do have some experience in bioinformatics with analysis of RNAseq, proteome or other tools using R. However, galaxy is new for me (a molecular biologist with some knowledge in bioinformatics) and having some step by step description on how to install VAPPER would have helped. The lack of instructions may discourage the use of the software by others. Since the authors indicate that deposition of the tool through galaxy toolshed was to help less experienced users, it did not help me much. Hence, some additional description in the manuscript or indication of a step by step protocol (e.g. as supplementary material) on how to access and install the VAPPER would be useful, including computer requirements etc.

R.
The galaxy toolshed is a shared repository with rules and procedures that surpass us as the authors. However, we have compiled a detailed protocol as supplementary file 1, where we provide a step-by-step description on how to install VAPPER both locally and using Galaxy. This user guide also includes a description of the program usage, commands examples, and instructions to test the installation using test data provided in the Github directory.

Reviewer #2: This paper reports a pipeline, VAPPER, for analysing VSG antigen profiles present in the genomes and transcriptomes of the important animal pathogens Trypanosoma congolense and T. vivax. VAPPER is well documented and executed, was easy to install and test for this review (on Mac OSX) and performed as advertised.

(Very) minor points:
line 73: Typo - missed space
line 114-115: Sentence needs rewording (missing 'are'?)

R.
We would like to thank reviewer 2 and confirm that these minor points have been addressed.

Close
