# Supplementary material for: VAPPER: High-throughput variant antigen profiling in African trypanosomes of livestock
Source: Gigascience. 2019 Aug 29;8(9):giz091. doi: 10.1093/gigascience/giz091 (PMC6735694; doi:10.1093/gigascience/giz091)
Supplement: giz091_Reviewer_1_Report_Original_Submission — Igor Cestari -- 1/3/2019 Reviewed [file giz091_reviewer_1_report_original_submission.pdf]

Reviewer Report

Title: VAPPER: High-throughput Variant Antigen Profiling in African trypanosomes of Livestock

Version: Original Submission **Date:** 1/3/2019

Reviewer name: Igor Cestari

Reviewer Comments to Author:

Summary:
VAPPER perform variant antigen profile (VPA) of multigenic variant surface glycoproteins (VSG) of trypanosomes of veterinary importance to quantify variant antigen diversity. The tool contains species-specific pipelines for analysis of genomes or transcriptomes of T. congolense or T. vivax strains. Data inputs are genome or transcriptome (the latter for T. congolense only), as trimmed paired sequencing reads (FASTQ) or assembled contig (FASTA) formats. For T. congolense, VAPPER searches for pre-defined protein motifs (characterized by the authors in previous publications) and defines phylotypes frequencies (within 15 phylotypes) as a basis for the identification and quantification of VAPs. For T. vivax, it uses a database with pre-defined VSG sequences which are somewhat distantly related due to the apparent lack of VSG gene recombination in this organism, and thus the VSG database is sufficient (perhaps comprehensive enough) to many T. vivax strains. The results are presented in tables of frequencies, heatmaps and PCA plots using standards outputs (HTML/pdf/png). VAPPER is available through galaxy or Linux.
General comments:
This is a useful tool for analysis of VSGs in trypanosomes of veterinary importance. The tool will support the analysis of VSG gene families which is challenging due to their abundance, mutation and/or recombination rates. A concern is VAPPER limitation to trypanosomes of veterinary importance, and thus the preliminary character of this work. It would be relevant to have it available to other trypanosomes, e.g. T. brucei brucei. Also, T. brucei that infect humans (e.g. T. brucei gambiense or rhodesiense), especially given the number of transcriptomes and genomes available for these parasites and the ability to track changes in VSG repertoire during infections with sequencing technologies. Also, it seems preliminary that the authors did not include validation of their tool with transcriptome of T. vivax, which is due to the lack of enough (as indicated by authors) transcriptome data. This work deserves to be published; however, it would be advantageous to include the option for analysis of variant antigens of other pathogens, at least the closest related T. brucei and additional validation to be complete.
Specific comments:
The limitation of the tool for two pathogens is discouraging, and I am astonished that the software did not include analysis of VSGs from T. brucei spp. which causes animal and human African trypanosomiasis. The authors also did not justify why they are not including an analysis of T. brucei spp. VSG genes since there is potential scientific interest from those working in the T. brucei field. Also, the implementation of VAPPER for analysis of other large antigen gene families such as those in T. cruzi, Leishmania, and Plasmodium etc was not included or discussed.
My suggestions are:

1) Authors should address why VAPPER does not analyze multigene families of other pathogens, and it should add the option to analyze (at least) T. brucei VSGs. The title of the manuscript and its introduction is suggestive of a broad utility of the tool for African trypanosomes, and not T. vivax and T. congolese only. This only became apparent much later in the text.
2) Validation of tVAP for T. vivax was not done due to the lack of transcriptomic data. How much data is needed for validation of this part of the tool? Because tVAP analysis for T. vivax uses a different pipeline than that of T. congolense, the validation of T. congolense does not guarantee that VAPPER would perform well for T. vivax tVAP. Can the authors generate the data to test the tool? This is another limitation of the tool which indicates that the work is still preliminary, and the authors should consider some additional effort to conclude this work prior publication. I do encourage the authors to do so, as VAPPER has the potential to be of interest in the parasitology community.
Minor comments:
1) The author should include in the introduction why is relevant to study the variant antigen profile of a pathogen and indicate examples. That VSGs are essential for immune evasion is clear, but it is not so clear the advantages (or when it would be necessary) to study the whole repertoire of VSGs and separate them in distinct clades (or groups). One possibility is that the analysis of variant antigen repertoires over an infection, which might provide insights into recombination mechanisms or other processes. Although some examples are included in the Results, it would be good to have examples of potential applications in the Introduction.
2) I found it difficult to install the tool using galaxy, and I did not find much instruction on how to proceed with this in the manuscript. I do have some experience in bioinformatics with analysis of RNAseq, proteome or other tools using R. However, galaxy is new for me (a molecular biologist with some knowledge in bioinformatics) and having some step by step description on how to install VAPPER would have helped. The lack of instructions may discourage the use of the software by others. Since the authors indicate that deposition of the tool through galaxy toolshed was to help less experienced users, it did not help me much. Hence, some additional description in the manuscript or indication of a step by step protocol (e.g. as supplementary material) on how to access and install the VAPPER would be useful, including computer requirements etc.

Level of Interest

Please indicate how interesting you found the manuscript: Choose an item.

Quality of Written English

Please indicate the quality of language in the manuscript: Choose an item.

Declaration of Competing Interests

Please complete a declaration of competing interests, considering the following questions:

- Have you in the past five years received reimbursements, fees, funding, or salary from an organisation that may in any way gain or lose financially from the publication of this manuscript, either now or in the future?
- Do you hold any stocks or shares in an organisation that may in any way gain or lose financially from the publication of this manuscript, either now or in the future?
- Do you hold or are you currently applying for any patents relating to the content of the manuscript?
- Have you received reimbursements, fees, funding, or salary from an organization that holds or has applied for patents relating to the content of the manuscript?
- Do you have any other financial competing interests?
- Do you have any non-financial competing interests in relation to this paper?

If you can answer no to all of the above, write 'I declare that I have no competing interests' below. If your reply is yes to any, please give details below.

I declare that I have no competing interests

I agree to the open peer review policy of the journal. I understand that my name will be included on my report to the authors and, if the manuscript is accepted for publication, my named report including any attachments I upload will be posted on the website along with the authors' responses. I agree for my report to be made available under an Open Access Creative Commons CC-BY license (http://creativecommons.org/licenses/by/4.0/). I understand that any comments which I do not wish to be included in my named report can be included as confidential comments to the editors, which will not be published.

I agree to the open peer review policy of the journal

To further support our reviewers, we have joined with Publons, where you can gain additional credit to further highlight your hard work (see: https://publons.com/journal/530/gigascience). On publication of this paper, your review will be automatically added to Publons, you can then choose whether or not to claim your Publons credit. I understand this statement.

Yes Choose an item.
